# Chronic Diseases as a Predictor for Severity and Mortality of COVID-19: A Systematic Review With Cumulative Meta-Analysis

**DOI:** 10.3389/fmed.2021.588013

**Published:** 2021-09-01

**Authors:** JinSong Geng, XiaoLan Yu, HaiNi Bao, Zhe Feng, XiaoYu Yuan, JiaYing Zhang, XiaoWei Chen, YaLan Chen, ChengLong Li, Hao Yu

**Affiliations:** ^1^Department of Medical Informatics, Medical School of Nantong University, Nantong, China; ^2^Department of Emergency Medicine, Affiliated Hospital of Nantong University, Nantong, China; ^3^Library and Reference Department, Zhejiang University School of Medicine First Affiliated Hospital, Hangzhou, China; ^4^Department of Population Medicine, Harvard Medical School and Harvard Pilgrim Health Care Institute, Boston, MA, United States

**Keywords:** chronic diseases, COVID-19, systematic review, cumulative meta-analysis, severity, mortality

## Abstract

**Introduction:** Given the ongoing coronavirus disease 2019 (COVID-19) pandemic and the consequent global healthcare crisis, there is an urgent need to better understand risk factors for symptom deterioration and mortality among patients with COVID-19. This systematic review aimed to meet the need by determining the predictive value of chronic diseases for COVID-19 severity and mortality.

**Methods:** We searched PubMed, Embase, Web of Science, and Cumulative Index to Nursing and Allied Health Complete to identify studies published between December 1, 2019, and December 31, 2020. Two hundred and seventeen observational studies from 26 countries involving 624,986 patients were included. We assessed the risk of bias of the included studies and performed a cumulative meta-analysis.

**Results:** We found that among COVID-19 patients, hypertension was a very common condition and was associated with higher severity, intensive care unit (ICU) admission, acute respiratory distress syndrome, and mortality. Chronic obstructive pulmonary disease was the strongest predictor for COVID-19 severity, admission to ICU, and mortality, while asthma was associated with a reduced risk of COVID-19 mortality. Patients with obesity were at a higher risk of experiencing severe symptoms of COVID-19 rather than mortality. Patients with cerebrovascular disease, chronic liver disease, chronic renal disease, or cancer were more likely to become severe COVID-19 cases and had a greater probability of mortality.

**Conclusions:** COVID-19 patients with chronic diseases were more likely to experience severe symptoms and ICU admission and faced a higher risk of mortality. Aggressive strategies to combat the COVID-19 pandemic should target patients with chronic diseases as a priority.

## Introduction

Coronavirus disease 2019 (COVID-19) is an infectious disease caused by severe acute respiratory syndrome coronavirus 2 (SARS-CoV-2). The COVID-19 outbreak was declared as a public health emergency of international concern by the World Health Organization (WHO) on January 30, 2020 (1). Since then, the disease has been spreading quickly around the world, reaching 9.296 million cases and 479,133 deaths as of June 25, 2020 (2). The ongoing COVID-19 pandemic has led to a rapidly growing demand for healthcare facilities and healthcare workers, leaving healthcare systems in many countries overstretched and unable to perform effectively (3).

The COVID-19 symptoms range from very mild to severe problems. While it was reported that the majority of COVID-19 cases were mild and required limited treatment (4), those patients with severe COVID-19 might need hospitalization or intensive care and have worse outcomes, such as death. Identifying risk factors for serious cases and mortality can be helpful in guiding public health interventions for protecting the most vulnerable groups of the population from COVID-19. For example, the risk factor information can be used to design risk stratification tools and clinical pathways, thus establishing more effective early intervention strategies and resource allocation policies.

COVID-19 is a serious global health threat, with more than 99% of confirmed cases currently coming from countries outside China. However, the vast majority of the published review articles relied almost exclusively on the studies conducted in China (5–11). In fact, several published reviews included data from only few countries outside China (12–14). Consequently, the limited information prevented decision-makers and patients from better recognizing the global evidence about risk factors for adverse COVID-19 outcomes. In addition, there is a serious concern about the validity and generalizability of the evidence on risk factors in COVID-19 patients generated by the published review articles, which failed to address the clinical heterogeneity of patients with COVID-19 among the observational studies. For instance, a meta-analysis (11) combined data from intensive care unit (ICU) admission and mortality into a single effect measure to find risk factors for progression of COVID-19, while another meta-analysis (8) pooled data from patients with severe COVID-19 symptoms and those who were admitted to the ICU into one group. Further systematic reviews are needed to address this issue of patient heterogeneity to improve the validity and generalizability of the evidence.

This paper aimed to fill the gap by conducting a systematic review with meta-analysis to determine the predictive value of chronic diseases for the severity and mortality of COVID-19. Our analysis examined global evidence to generate systematic and robust findings. To our knowledge, this study represented the most comprehensive meta-analysis of COVID-19 severity, mortality, ICU admission, and acute respiratory distress syndrome (ARDS). Furthermore, it was the first study to determine the associations between several chronic conditions, including obesity, asthma, and hyperlipidemia, with clinical outcomes of COVID-19 patients. We also included only studies from the peer-reviewed journals to ensure the validity of conclusions, while some meta-analyses used manuscripts in preprint servers to increase the sample size (9, 15–17).

## Methods

Methods for this systematic review were developed according to the recommendations from the MOOSE statement (18) and PRISMA statement (19) for reporting of systematic review and meta-analysis.

### Criteria for Considering Studies for This Review

Observational studies that focused on adult patients (aged over 16 years) with COVID-19 and investigated the association between chronic diseases and severity, ICU admission, mortality, and ARDS of COVID-19 were included.

The following types of studies were excluded: (1) studies that only included infants, children, and pregnant women; (2) studies that only included decedents (only death patients were enrolled in each group); (3) studies that did not classify patients into different groups by severity, type of hospital wards (i.e., general wards, ICU), mortality, or ARDS; (4) studies that did not have enough statistical information to be extracted from each group of patients; (5) duplicated publication of the same research results, i.e., data from the same hospitals within the same period; and (6) descriptive reviews, systematic review, meta-analysis, opinion, editorial, comments, and conference abstracts without full article publication.

### Study Outcomes

The primary outcome measure was the association between chronic diseases and the severity of COVID-19 patients. Secondary outcomes included the association between chronic diseases and mortality, ICU admission, and ARDS of COVID-19 hospitalized patients. The chronic diseases in our review were hypertension, diabetes, pulmonary disease [chronic obstructive pulmonary disease (COPD), asthma, and unspecified type], cardiovascular disease (coronary heart disease, heart failure, and unspecified type), cerebrovascular disease, hyperlipidemia, obesity, chronic liver disease, chronic renal disease, cerebrovascular disease, and cancer. The association between Charlson comorbidity index and the clinical outcomes of COVID-19 patients was also analyzed.

### Search Strategy

Studies were identified by searching PubMed, Embase, Science Citation Index Expanded (Web of Science), and Cumulative Index to Nursing and Allied Health (CINAHL) Complete. Our search strategy is listed in Appendix 1 in Supplementary Material. References from the retrieved papers were also searched. Studies published between December 1, 2019, and December 31, 2020 were included.

### Study Selection and Data Extraction

In accordance with the defined inclusion criteria, two reviewers independently read the title and abstract of each study retrieved by the search. The reviewers excluded studies that did not meet the inclusion criteria. After screening the title and abstract of each study, the full texts of eligible citations were then assessed by the two reviewers independently.

A third reviewer was consulted when the two reviewers could not agree on selecting a study. The reviewers developed a data extraction form and used it to extract data to reflect the characteristics of each included study. If the data from the same hospitals in the same period were published several times, only the paper with the largest sample size was included.

The included studies varied in their classification of disease severity, ranging from mild, moderate, severe, to critical severe. We categorized mild and moderate cases into the non-severe group and severe and critical severe cases into the severe group. We considered the following cases as the ICU groups—ICU admission and requiring invasive mechanical ventilation, and critical cases of illness that were admitted to the ICU.

### Risk of Bias Assessment

We used the tools developed by the Joanna Briggs Institute (JBI) (20–23) to assess the risk of bias of the included studies. The JBI critical appraisal tools for cohort studies, case series, case–control studies, and cross-sectional studies included 11, 10, 10, and 8 items, respectively. The appraisal tools addressed the internal validity and risk of bias of the study design, particularly confounding, selection, and information bias, in addition to the importance of clear reporting.

### Statistical Analysis

We conducted a meta-analysis when data from more than one study could be combined. We calculated pooled estimates of odds ratio (OR) and 95% confidence interval (CI) by the generic inverse variance method using STATA 14.2 (STATA Corporation, College Station, TX, USA). We tested the heterogeneity of effective measures using the *I*^2^ statistic. We defined *I*^2^ values greater than 50% as considerable or substantial heterogeneity (24). For data with substantial heterogeneity, a random-effects model using the method developed by DerSimonian and Laird (25) was specified to address heterogeneity among the studies. For data with unsubstantial heterogeneity, a fixed-effects model with the inverse variance method was used to synthesize the data.

For the most prevalent chronic diseases including hypertension, diabetes, COPD, coronary heart disease, cerebrovascular disease, and cancer, we conducted the cumulative meta-analysis according to the season of admission of the patients and the increasing sample size of the included studies within each season. A cumulative meta-analysis is helpful to assess the dynamics of how the summary results change with a newly added study (26). R 4.0.3 (The R Foundation for Statistical Computing, Vienna, Austria) was used to conduct a cumulative meta-analysis.

## Results

### Literature Search and Study Selection

**Figure 1:**
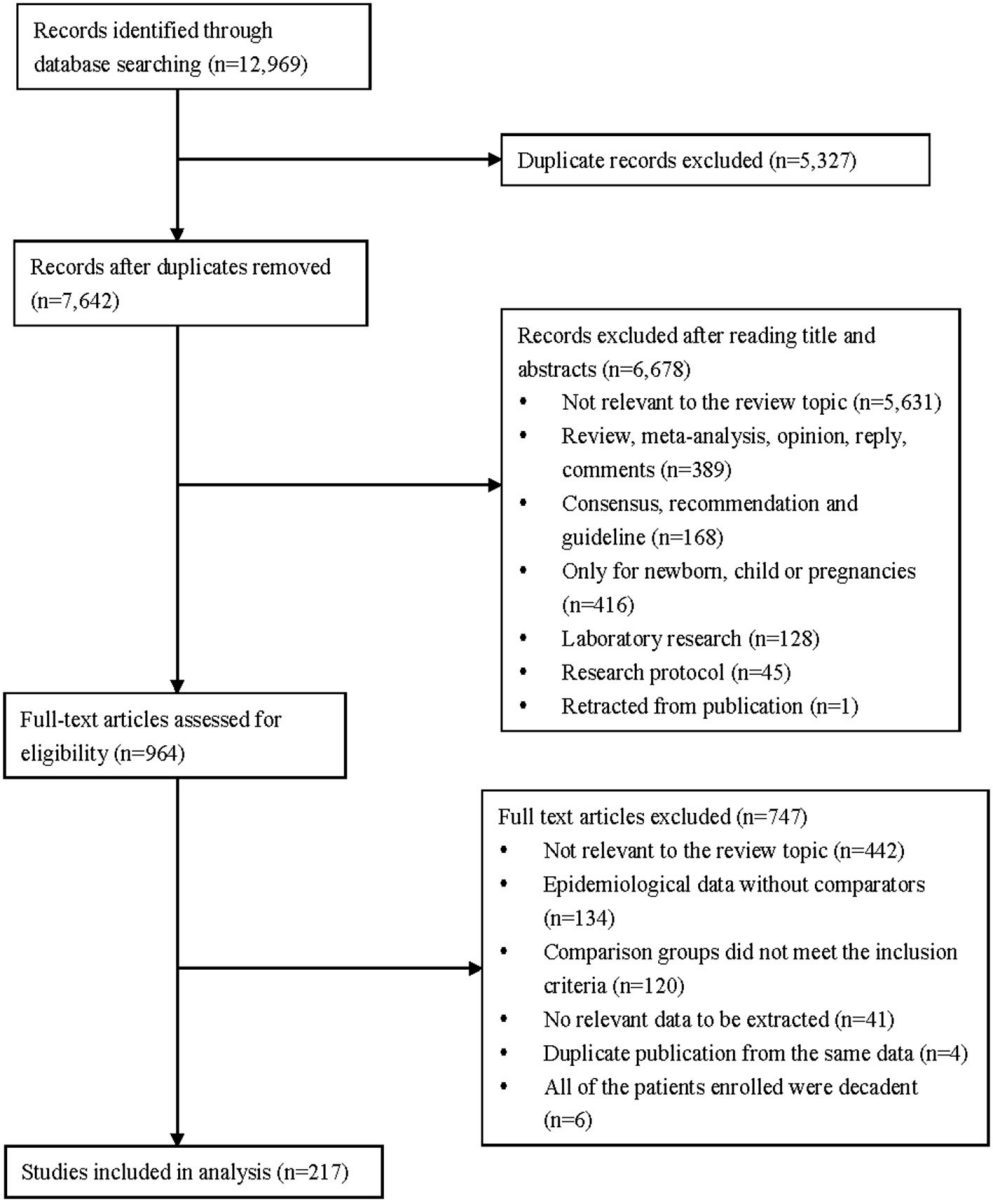
Flow diagram of the study selection for inclusion in the systematic review.

Two hundred and seventeen observational studies (27–243) with 624,986 patients met the inclusion criteria in our systematic review. A PRISMA flowchart summarized our search results and study selection procedure (Figure 1).

### Characteristics and Quality of the Included Studies

Table 1 presents the characteristics of the 217 included studies. The studies were carried out in 26 countries, 83 (38.25%) of them were performed in multicenters, 123 (56.68%) were case series, and 82 (37.79%) were cohort studies. The date of admission of the patients was from December 11, 2019, to August 1, 2020. Most of the outcome variables were about mortality (48.39%) and severity (34.10%). Details of the characteristics are shown in Appendix 2 in Supplementary Material.

**Table 1:** Characteristics of the included studies.

**Characteristics**	**Number of studies**	**Percentage of the included studies (%)**
**Country**		
China	111	51.15
USA	21	9.68
Italy	20	9.22
South Korea	7	3.23
Spain	7	3.23
Iran	6	2.76
Germany	5	2.30
France	5	2.30
Mexico	5	2.30
India	4	1.84
UK	4	1.84
Brazil	3	1.38
Congo	2	0.92
Kuwait	2	0.92
Saudi Arabia	2	0.92
Bangladesh	1	0.46
Bolivia	1	0.46
Bulgaria	1	0.46
Denmark	1	0.46
Greece	1	0.46
Ireland	1	0.46
Oman	1	0.46
Poland	1	0.46
Qatar	1	0.46
Switzerland	1	0.46
Turkey	1	0.46
Multicountries^a^	2	0.92
**Data source**		
Multicenters	83	38.25
Single center	134	61.75
**Type of study**		
Case series	123	56.68
Retrospective cohort study	71	32.72
Prospective cohort study	10	4.61
Ambispective cohort study	1	0.46
Case–control study	4	1.84
Cross-sectional study	8	3.69
**Type of comparisons^b^**		
Severe vs. non-severe	74	34.10
Death vs. survival	105	48.39
ICU vs. non-ICU	53	24.42
ARDS vs. non-ARDS	6	2.76

a *One was carried out in Europe, and the other was conducted in China, Europe, and North America*.

b *Seventeen studies had two types of comparisons. Two studies had three types of comparisons*.

The quality assessment score for the case series ranged from 6 to 10 (9.13 ± 0.84) with 102 of them (82.93%) having a score higher than 8 (10 indicates the best quality). Among the case series, 48 studies (39.02%) did not indicate a consecutive inclusion of patients, and 45 studies (36.59%) did not have a complete inclusion of patients.

The score of cohort studies ranged from 6 to 11 (10.04 ± 0.92) with 62 cohort studies (75.61%) having a score more than 9 (11 indicates the best quality). Length of follow-up was not mentioned in 34 cohort studies, and the reasons for losses to follow-up were not described in 6 studies. In addition, we were not sure whether patients across different centers had similar characteristics in relation to exposure (27 studies). Appendix 3 in Supplementary Material presents details of the risk of bias assessment.

### Association Between Chronic Diseases and Severity of COVID-19

We identified 74 studies of COVID-19 severity, involving a total of 32,213 patients and 8,433 cases of severe COVID-19. Among these studies, 65 of them were performed in China, 3 in the USA, and 1 in Bulgaria, Congo, Kuwait, Saudi Arabia, South Korea, and Spain, respectively.

As shown in Table 2, the prevalence of patients with any type of chronic disease in the group of severe COVID-19 was substantially higher than that of the non-severe group (60.71 vs. 31.81%; OR 3.70, 95% CI 2.98–4.61). Hypertension (OR 3.05, 95% 2.60–3.59), diabetes (OR 2.55, 95% CI 2.14–3.03), COPD (OR 3.91, 95% CI 3.05–5.02), asthma (OR 1.93, 95% CI 1.53–2.42), unspecified type of pulmonary disease (OR 2.48, 95% CI 2.03–3.03), coronary heart disease (OR 2.04, 95% CI 1.72–2.42), unspecified type of cardiovascular disease (OR 3.01, 95% CI 2.64–3.43), cerebrovascular disease (OR 2.32, 95% CI 1.83–2.94), obesity (OR 2.63, 95% CI 1.70–4.07), chronic liver disease (OR 1.96, 95% CI 1.64–2.35), chronic renal disease (OR 2.09, 95% 1.52–2.87), and cancer (OR 2.33, 95% 1.90–2.87) were all associated with significantly higher risk of severity among COVID-19 patients. There were only two types of the study chronic diseases (i.e., heart failure and hyperlipidemia) that were not significantly associated with the severity of COVID-19 (*P* > 0.05). However, the prevalence of heart failure or hyperlipidemia was only reported in three studies.

**Table 2:** Associations between chronic diseases and severity of COVID-19.

**Chronic diseases**	**Number of included studies**	**Number of severe COVID-19 patients**	**Number of non-severe COVID-19 patients**	**Prevalence in the severe group (%)**	**Prevalence in the non-severe group (%)**	***I*** **^2^**	**Pooled OR** **(95% CI)**	***P*** **-value**
Hypertension	67	6,453	18,352	42.97	20.04	76.7	3.05 (2.60–3.59)	0.000
Diabetes	70	7,184	21,106	20.98	10.78	67.7	2.55 (2.14–3.03)	0.000
**Pulmonary diseases**								
COPD	33	4,571	11,949	4.11	1.13	0.0	3.91 (3.05–5.02)	0.000
Asthma (conclusion changed)	9	997	3,671	14.24	13.54	0.0	1.93 (1.53–2.42)	0.000
Unspecified type^a^	20	1,720	5,917	18.84	16.48	5.7	2.48 (2.03–3.03)	0.000
**Cardiovascular diseases**								
Coronary heart disease	20	3,184	9,133	9.58	4.03	34.7	2.04 (1.72–2.42)	0.000
Heart failure	3	83	100	14.46	14.00	57.6	1.74 (0.26–11.45)	0.567
Unspecified type^a^	32	3,052	9,158	21.04	8.83	45.9	3.01 (2.64–3.43)	0.000
Cerebrovascular disease	25	3,085	10,306	4.64	1.85	19.5	2.32 (1.83–2.94)	0.000
Hyperlipidemia	3	222	315	2.70	2.54	0.0	1.01 (0.34–3.02)	0.985
Obesity	9	1,075	2,025	18.14	8.74	63.1	2.63 (1.70–4.07)	0.000
Chronic liver disease	27	4,176	12,622	8.19	9.55	26.4	1.96 (1.64–2.35)	0.000
Chronic renal disease	33	5,198	15,388	4.66	2.56	50.2	2.09 (1.52–2.87)	0.000
Cancer	40	3,507	10,917	5.47	2.56	6.6	2.33 (1.90–2.87)	0.000
Any types of chronic disease^a^	33	3,118	8,206	60.71	31.81	71.4	3.70 (2.98–4.61)	0.000

a *The included studies did not mention the specific types of disease in this category*.

Appendix 4 in Supplementary Material presents the forest plots of cumulative meta-analysis for major types of chronic diseases. Subsequent studies increased the precision of the point estimate, and no change occurred in the direction of the effect size.

### Association Between Chronic Diseases and Mortality of COVID-19

We found that 105 studies with a total of 350,522 patients and 68,157 deaths presented data on mortality. Among the studies, 40 of them were carried out in China, 12 in Italy, 11 in the USA, 6 in Iran, 6 in Spain, 4 in India, 4 in the UK, 3 in Brazil, 3 in South Korea, 2 in France, 2 in Mexico, and 1 in Bangladesh, Bolivia, Congo, German, Greece, Ireland, Kuwait, Saudi Arabia, Switzerland, and Turkey, respectively. We also found two studies that were conducted in multicountries.

As shown in Table 3, 36.49% of patients who died had at least one type of chronic disease. Hyperlipidemia (52.80%) was the most common chronic condition among patients who died, followed by hypertension (37.53%) and unspecified type of cardiovascular disease (28.56%). Hypertension (OR 2.31, 95% CI 2.04–2.61), diabetes (OR 1.99, 95% CI 1.82–2.18), COPD (OR 2.95, 95% CI 2.48–3.50), unspecified type of pulmonary disease (OR 2.05, 95% CI 1.83–2.31), coronary heart disease (OR 2.46, 95% CI 2.14–2.82), heart failure (OR 2.74, 95% CI 2.21–3.40), unspecified type of cardiovascular disease (OR 2.59, 95% CI 2.24–3.00), cerebrovascular disease (OR 2.46, 95% CI 2.08–2.91), hyperlipidemia (OR 1.72, 95% CI 1.07–2.77), chronic liver disease (OR 1.52, 95% CI 1.30–1.77), chronic renal disease (OR 2.85, 95% CI 2.44–3.33), and cancer (OR 2.11, 95% CI 1.85–2.42) were associated with a higher risk of mortality.

**Table 3:** Associations between chronic diseases and mortality of COVID-19.

**Chronic diseases**	**Number of included studies**	**Number of COVID-19 deaths**	**Number of COVID-19 survivors**	**Prevalence in the death group (%)**	**Prevalence in the survival group (%)**	***I*** **^2^**	**Pooled OR** **(95% CI)**	***P*** **-value**
Hypertension	96	49,072	205,854	37.53	26.86	92.2	2.31 (2.04–2.61)	0.000
Diabetes	105	57,121	250,467	22.34	15.29	84.8	1.99 (1.82–2.18)	0.000
**Pulmonary diseases**								
COPD	54	13,013	52,915	13.18	6.93	73.0	2.95 (2.48–3.50)	0.000
Asthma	16	11,026	36,444	8.51	8.50	0.0	0.74 (0.68–0.80)	0.000
Unspecified type^a^	29	19,961	120,999	21.91	13.72	63.1	2.05 (1.83–2.31)	0.000
**Cardiovascular diseases**								
Coronary heart disease	35	9,533	36,597	22.36	11.73	63.1	2.46 (2.14–2.82)	0.000
Heart failure	24	18,504	125,276	21.17	6.99	92.9	2.74 (2.21–3.40)	0.000
Unspecified type^a^	52	16,043	52,536	28.56	12.15	79.3	2.59 (2.24–3.00)	0.000
Cerebrovascular disease	40	16,889	122,332	11.82	4.23	69.7	2.46 (2.08–2.91)	0.000
Hyperlipidemia	6	9,875	69,330	52.80	29.94	97.8	1.72 (1.07–2.77)	0.000
Obesity	27	35,778	119,672	7.00	8.77	93.0	1.19 (0.94–1.51)	0.147
Morbid obesity	3	1,556	3,975	9.51	10.06	0.0	0.98 (0.80–1.20)	0.858
Chronic liver disease	27	9,988	26,493	2.93	2.30	32.3	1.52 (1.30–1.77)	0.000
Chronic renal disease	60	23,024	82,836	14.11	5.68	78.3	2.85 (2.44–3.33)	0.000
Cancer	65	26,704	142,413	11.58	5.66	70.5	2.11 (1.85–2.42)	0.000
**Charlson index**								
0	3	34,308	129,484	78.54	83.17	99.3	0.31 (0.18–0.51)	0.000
1	2	23,103	97,763	4.79	7.06	98.1	1.25 (0.67–2.33)	0.481
≥2	3	34,308	129,484	20.29	10.95	98.7	4.22 (2.56–6.96)	0.000
Any type of chronic diseases^a^	43	35,905	113,002	36.49	24.88	88.8	3.11 (2.64–3.65)	0.000

a *The included studies did not mention the specific type of disease in this category*.

We found no significant correlation between obesity (OR 1.19, 95% CI 0.94–1.51) and death. We also did the subgroup analysis for morbid obesity (BMI ≥ 40 kg/m^2^), and the results were not statistically significant (OR 0.98, 95% CI 0.80–1.20). Our meta-analysis showed that asthma was associated with a reduced risk of mortality (OR 0.74, 95% CI 0.68–0.80). The Charlson index score equals to 0 seemed to be a protective factor for mortality (OR 0.31, 95% CI 0.18–0.51), while a score ≥2 might be consistent with the higher likelihood of death (OR 4.22, 95% CI 2.56–6.96).

The cumulative meta-analysis showed that the sample size increased; the CI for hypertension, diabetes, COPD, coronary heart disease, and cancer became increasingly narrower; and statistical significance was more common (Appendix 4 in Supplementary Material). However, the subgroup of cerebrovascular disease with the admission date of patients in summer was not significant (*P* > 0.05), probably due to only two studies were included.

### Association Between Chronic Diseases and ICU Admission of COVID-19

Fifty-three studies involving a total of 260,465 patients and 12,233 cases of ICU admission were included. Of these studies, 14 were conducted in China; 9 in the USA; 6 in Italy; 4 in South Korea; 3 in France, Germany, and Mexico, respectively; 2 in Kuwait; and 1 in Denmark, India, Iran, Oman, Poland, Qatar, Saudi Arabia, Spain, and Turkey, respectively.

We found that 73.62% of the ICU patients had at least one type of chronic disease, which was significantly higher than that in the non-ICU group (OR 2.82, 95% CI 2.23–3.56) (Table 4). Hypertension (OR 2.24, 95% CI 1.90–2.63), diabetes (OR 2.50, 95% CI 2.18–2.87), COPD (OR 2.76, 95% CI 1.99–3.82), unspecified type of pulmonary disease (OR 1.40, 95% CI 1.26–1.56), coronary heart disease (OR 2.16, 95% CI 1.56–2.99), heart failure (OR 1.80, 95% CI 1.44–2.55), unspecified type of cardiovascular disease (OR 2.38, 95% CI 1.92–2.96), hyperlipidemia (OR 1.53, 95% CI 1.22–1.93), obesity (OR 1.86, 95% CI 1.49–2.31), chronic renal disease (OR 2.25, 95% CI 1.73–2.94), and cancer (OR 1.57, 95% CI 1.39–1.77) were significant predictive factors for admission to ICU. On the other hand, asthma and chronic liver disease were not significantly associated with ICU admission (*P* > 0.05). The association between cerebrovascular disease and ICU admission was not very obvious (*P* = 0.048).

**Table 4:** Associations between chronic diseases and ICU admission of COVID-19.

**Chronic diseases**	**Number of included studies**	**Number of ICU COVID-19 patients**	**Number of non-ICU COVID-19 patients**	**Prevalence in the ICU group (%)**	**Prevalence in the non-ICU group (%)**	***I*** **^2^**	**Pooled OR** **(95% CI)**	***P*** **-value**
Hypertension	50	12,062	247,158	47.78	24.72	87.1	2.24 (1.90–2.63)	0.000
Diabetes	51	12,188	247,334	37.27	17.28	79.6	2.50 (2.18–2.87)	0.000
**Pulmonary disease**								
COPD	25	7,648	223,993	6.85	2.43	69.7	2.76 (1.99–3.82)	0.000
Asthma	11	7,070	220,430	3.92	3.02	26.1	1.07 (0.94–1.22)	0.280
Unspecified type^a^	14	2,690	18,052	20.86	16.62	0.0	1.40 (1.26–1.56)	0.000
**Cardiovascular disease**								
Coronary heart disease	20	2,743	19,716	24.32	14.09	79.9	2.16 (1.56–2.99)	0.000
Heart failure	11	3,969	24,536	20.58	11.81	62.7	1.80 (1.44–2.25)	0.000
Unspecified type^a^	28	7,801	212,550	17.13	3.00	54.8	2.38 (1.92–2.96)	0.000
Cerebrovascular disease	19	2,587	20,957	10.86	4.67	80.7	1.66 (1.00–2.75)	0.048
Hyperlipidemia	4	1,713	11,354	54.64	51.77	53.2	1.53 (1.22–1.93)	0.000
Obesity	19	10,016	232,568	30.65	20.52	89.4	1.86 (1.49–2.31)	0.000
Chronic liver disease	16	889	6,699	2.92	1.97	0.0	1.48 (0.95–2.29)	0.082
Chronic renal disease	31	10,550	237,131	11.54	3.63	82.8	2.25 (1.73–2.94)	0.000
Cancer	30	3,286	21,949	14.39	11.93	34.2	1.57 (1.39–1.77)	0.000
Any type of chronic disease^a^	22	8,329	223,276	73.62	47.79	81.0	2.82 (2.23–3.56)	0.000

a *The included studies did not mention the specific type of disease in this category*.

The cumulative meta-analysis showed that the statistical significance of hypertension, diabetes, COPD, coronary heart disease, and cancer had the tendency of becoming evident with increasing sample size (Appendix 4 in Supplementary Material).

### Association Between Chronic Diseases and ARDS of COVID-19

Six studies involving a total of 2,128 patients and 635 cases of ARDS admission were included. Two of them were conducted in China, two in Italy, and 1 in Germany and the USA, respectively.

We found a significant association between hypertension (OR 2.17, 95% CI 1.78–2.66), diabetes (OR 2.32, 95% CI 1.70–3.17), coronary heart disease (OR 1.96, 95% CI 1.32–2.92), unspecified type of cardiovascular disease (OR 2.35, 95% CI 1.24–4.47), obesity (OR 2.25, 95% CI 1.18–4.28), chronic renal disease (OR 1.63, 95% CI 1.14–2.33), and occurrence of ARDS (Table 5). COPD, heart failure, and cerebrovascular disease were not significantly correlated with the risk of ARDS (*P* > 0.05).

**Table 5:** Associations between chronic diseases and ARDS of COVID-19.

**Chronic diseases^a^**	**Number of included studies**	**Number of COVID-19 patients with ARDS**	**Number of COVID-19 patients without ARDS**	**Prevalence in the ARDS group (%)**	**Prevalence in the non-ARDS group (%)**	***I*** **^2^**	**Pooled OR** **(95% CI)**	***P*** **-value**
Hypertension	6	635	1,493	58.11	38.71	0.0	2.17 (1.78–2.66)	0.000
Diabetes	6	635	1,493	16.54	9.58	21.5	2.32 (1.70–3.17)	0.000
COPD	4	499	986	15.23	8.01	68.4	1.35 (0.54–3.37)	0.516
**Cardiovascular disease**								
Coronary heart disease	2	449	856	12.03	6.43	0.0	1.96 (1.32–2.92)	0.001
Heart failure	2	449	856	10.69	5.26	58.4	1.85 (0.834–4.11)	0.130
Unspecified type^b^	3	162	611	10.49	7.20	0.0	2.35 (1.24–4.47)	0.009
Cerebrovascular disease	3	473	882	12.69	6.35	71.5	1.41 (0.46–4.30)	0.546
Obesity	4	525	1,272	28.95	12.42	70.7	2.25 (1.18–4.28)	0.014
Chronic renal disease	4	499	986	12.63	7.71	14.1	1.63 (1.14–2.33)	0.008

a *Only data for these chronic diseases were available to conduct meta-analyses*.

b *The included studies did not mention the specific type of disease in this category*.

## Discussion

Given the ongoing COVID-19 pandemic and the consequent global healthcare crisis, there is an urgent need to better understand risk factors for symptom deterioration and identify the vulnerable populations at higher risk for COVID-19 mortality. Our meta-analysis aimed to meet the need by examining global evidence, including 217 studies from 26 countries with 624,986 COVID-19 patients. Compared with a prior meta-analysis that showed that among COVID-19 patients, 20.3% required ICU admission and 32.8% had ARDS (244), our analysis provided further data on the association between chronic diseases and the different clinical prognoses of COVID-19 patients. According to our findings, COVID-19 patients with chronic diseases were more likely to have severe symptoms, ICU admissions, and an increased risk of mortality.

On the contrary, a meta-analysis found that pre-existing chronic conditions were not correlated with COVID-19 mortality (OR 2.09, 95% CI 0.26 to 16.67) (12). However, the conclusion of that meta-analysis was not reliable due to the fact that it only included three studies with a small sample size (453). In comparison, our meta-analysis had a much large sample size and identified the significant associations between a variety of chronic conditions and COVID-19 mortality, such as hypertension, diabetes, COPD, unspecified type of pulmonary disease, coronary heart disease, heart failure, unspecified type of cardiovascular disease, cerebrovascular disease, hyperlipidemia, chronic liver disease, chronic renal disease, and cancer.

We found that among COVID-19 patients, hypertension was a common comorbidity and was associated with COVID-19 severity, ICU admission, ARDS, and mortality. We found that COPD was the strongest predictive comorbidity for COVID-19 severity, ICU admission, and mortality, a finding that is consistent with prior research results, confirming that COPD patients are particularly vulnerable for very severe or critical COVID-19 cases (6).

Whereas, the published COVID-19 systematic reviews used the term “cardiovascular disease” generally (11, 13, 245), merely merged different types of cardiovascular diseases into a single measure, or only used the data from “unspecified type of cardiovascular diseases” as the outcome (6, 11, 13), we categorized cardiovascular diseases into three groups—coronary heart disease, heart failure, and unspecified type of cardiovascular diseases—to provide specific evidence for decision-makers. We found that coronary heart disease was a potential risk factor for the severity, ICU admission, mortality, and ARDS of COVID-19, while heart failure could increase the probability of ICU admission and mortality.

According to our results, cerebrovascular disease was an important comorbidity for COVID-19 mortality. We also identified cerebrovascular disease as a risk factor for severity of COVID-19 patients, which was consistent with other meta-analyses (14, 245). However, we found that there was a weak association between cerebrovascular disease and the risk of ICU admission, a finding that was different from a meta-analysis showing that cardio-cerebrovascular diseases were about 3-fold higher in ICU patients than in their non-ICU counterparts (17). However, only six studies were included in that meta-analysis, and its method was problematic as it calculated relative risk despite the fact that it included retrospective studies. Furthermore, cardiovascular disease and cerebrovascular disease were combined into a single outcome measure in the previous meta-analysis.

Asthma is a chronic disease of the air passages of the lungs which inflames and narrows them. Both its prevalence and mortality increased in recent decades, accounting for 272.68 million cases (3.57%) and 0.49 million deaths (0.006%) in the year 2017 (246). A multicenter retrospective study in 10 US hospitals found that asthma did not lead to an increased risk of hospitalization (RR 0.96, 95% CI 0.77–1.19) for COVID-19 patients after adjusting for age, sex, gender, and comorbidities (247). However, we found that asthma was associated with the severity of COVID-19 but tended to become a protective factor to reduce mortality risk. On the other hand, a published meta-analysis demonstrated that asthma patients were not predisposed to severe COVID-19 infections (248). It should be noted that the meta-analysis searched for articles published from January 1, 2020, to August 28, 2020, which was even shorter than our study, and only five studies were included in that meta-analysis.

The prevalence of obesity in many countries has been increasing rapidly in recent decades. We found that obese patients were at a higher risk of developing severe COVID-19 symptoms. However, the association between obesity (BMI ≥ 28 or 30 kg/m^2^) and mortality was not statistically significant. We did not find a significant relationship between morbid obesity (BMI ≥ 40 kg/m^2^) and mortality.

Hyperlipidemia involves an imbalance of cholesterol levels, including low-density lipoprotein cholesterol (LDL-C) and high-density lipoprotein cholesterol (HDL-C) in the blood. It has become common in many countries (249), especially in the USA where low HDL-C among adults aged 20 and over was 17.2% (250) and roughly 53% of adults had elevated LDL-C levels (251). Our results showed that hyperlipidemia was associated with increased ICU admission and mortality of COVID-19 patients.

A meta-analysis revealed an insignificant correlation between the increased risk of severe COVID-19 and liver disease, cancer, or renal disease (245). The insignificant results were probably due to the small number of studies included in the analysis—only five studies were included and all of them were from China. In contrast, our large sample size from multiple countries enabled us to find that those COVID-19 patients with chronic liver disease, cancer, or chronic renal disease were more likely to become severe cases and had a higher risk of mortality.

Our results emphasize the need for enhanced vigilance, priority for detection and testing, and aggressive COVID-19 therapy for patients with chronic diseases. Given our findings that COVID-19 patients with various chronic diseases were more likely to experience severe symptoms and ICU admissions and faced a higher risk of mortality, policymakers across different countries need to target patients with chronic diseases as a priority of their strategies to combat the COVID-19 pandemic. In particular, measures should be taken to protect the vulnerable groups with specific types of chronic disease, such as hypertension, diabetes, cardiovascular disease, and hyperlipidemia, each of which has a high prevalence in the general population.

For some of the less common chronic conditions, a targeted and intensive health protection strategy is also warranted. For example, although COPD is a less common condition among the general population, our analysis indicated that it is strongly associated with COVID-19 severity, ICU admission, and mortality. We also found that cerebrovascular disease, a less common condition that is the leading cause of serious long-term disability, was a significant comorbidity predicting mortality in COVID-19 patients. Thus, patients with COPD and/or cerebrovascular diseases should receive special attention from both policymakers and healthcare professionals.

Finally, our analysis suggested that more adequately powered studies should be conducted to investigate how the severity and mortality of COVID-19 are associated with morbid obesity and hyperlipidemia, and a composite measure of comorbidity such as the Charlson comorbidity index must be utilized. The risk factors for ARDS in patients with severe COVID-19 are also worthy of further analysis in the future.

The results of our systematic review should be interpreted in the context of its limitations. First, we did not include studies that only analyzed children, pregnancies, and healthcare professionals in order to ensure the homogeneity and representativeness of the general population. The existing systematic reviews found that children seemed to have a milder disease course and better prognosis than adults (252) and that vertical transmission of COVID-19 from pregnancies to newborns could not be ruled out (253). Second, there was a limited sample size on risk factors for ARDS, and future observational studies are still needed on this topic. Third, the predictive value of concurrent multiple chronic diseases for the prognosis of COVID-19 patients remains unclear. Fourth, we were unable to conduct subgroup analysis according to community dwellings and institutionalized individuals due to a lack of data from the included studies. The association between chronic diseases and severity of COVID-19 should be further analyzed in community care and institutional care, respectively. Finally, further observational studies and meta-analyses are still needed to explore the impacts of chronic diseases on the severity and mortality in later waves of the COVID-19 pandemic.

## Data Availability Statement

The original contributions presented in the study are included in the article/Supplementary Material, further inquiries can be directed to the corresponding author.

## Author Contributions

HY and JG designed the protocol. JG, XC, and CL performed the literature search and screening. XYu, HB, ZF, JZ, and XC extracted the data and did the quality assessment. JG and YC checked the data. XYuan took part in the interpretation of the data. JG and HY contributed to the meta-analysis and interpretation of the results and drafted the manuscript. All authors contributed to the article and approved the submitted version.

## Conflict of Interest

The authors declare that the research was conducted in the absence of any commercial or financial relationships that could be construed as a potential conflict of interest.

## Publisher's Note

All claims expressed in this article are solely those of the authors and do not necessarily represent those of their affiliated organizations, or those of the publisher, the editors and the reviewers. Any product that may be evaluated in this article, or claim that may be made by its manufacturer, is not guaranteed or endorsed by the publisher.
