# Supplementary material for: Chronic Diseases as a Predictor for Severity and Mortality of COVID-19: A Systematic Review With Cumulative Meta-Analysis
Source: Front Med (Lausanne). 2021 Sep 1;8:588013. doi: 10.3389/fmed.2021.588013 (PMC8440884; doi:10.3389/fmed.2021.588013)
Supplement: Supplementary file 1 [file Data_Sheet_1.pdf]

Supplementary Material

Appendix 1. Search strategy

PubMed

#1 "severe acute respiratory syndrome coronavirus 2" [Supplementary Concept] OR "COVID-19" [Supplementary Concept]

#2 "2019 nCoV"[Title/Abstract] OR "severe acute respiratory syndrome coronavirus 2"[Title/Abstract] OR "COVID-19"[Title/Abstract] OR "coronavirus disease 2019"[Title/Abstract] OR "novel coronavirus"[Title/Abstract] OR "new coronavirus"[Title/Abstract] OR "SARS-CoV-2"[Title/Abstract]

#3 feature[Title/Abstract] OR characteristic*[Title/Abstract] OR prevalence[Title/Abstract] OR symptom*[Title/Abstract] OR surviv*[Title/Abstract] OR death[Title/Abstract] OR die[Title/Abstract] OR mortality[Title/Abstract] OR mild[Title/Abstract] OR moderate[Title/Abstract] OR critical*[Title/Abstract]
#4 co-morbidit* OR comorbidit* OR complication OR chronic OR hypertension OR diabetes OR cancer OR tumor OR 'Chronic obstructive pulmonary disease' OR 'COPD' OR 'Acute respiratory distress syndrome' OR 'ARDS' OR cardiovascular OR cerebrovascular OR liver OR kidney OR brain OR heart OR stroke OR obesity

#5 (#1 OR #2) AND #3 AND #4

#6 Filter applied: observational study, case reports, comparative study

Embase

#1 'coronavirus disease 2019'/exp OR 'severe acute respiratory syndrome coronavirus 2'/exp OR '2019 ncov':ti,ab,kw OR 'severe acute respiratory syndrome coronavirus 2':ti,ab,kw OR 'covid 19':ti,ab,kw OR 'coronavirus disease 2019':ti,ab,kw OR 'novel coronavirus':ti,ab,kw OR 'new coronavirus':ti,ab,kw OR 'sars-cov-2':ti,ab,kw

#2 feature:ti,ab,kw OR characteristic*:ti,ab,kw OR prevalence:ti,ab,kw OR symptom*:ti,ab,kw OR predict*:ti,ab,kw OR severity:ti,ab,kw OR severe:ti,ab,kw OR surviv*:ti,ab,kw OR death:ti,ab,kw OR die:ti,ab,kw OR mortality:ti,ab,kw OR mild:ti,ab,kw OR moderate:ti,ab,kw OR critical*:ti,ab,kw

#3 'co-morbidit*':ti,ab,kw OR comorbidit*:ti,ab,kw OR complication:ti,ab,kw OR chronic:ti,ab,kw OR hypertension:ti,ab,kw OR diabetes:ti,ab,kw OR cancer:ti,ab,kw OR tumor:ti,ab,kw OR 'chronic obstructive pulmonary disease':ti,ab,kw OR 'copd':ti,ab,kw OR 'acute respiratory distress syndrome':ti,ab,kw OR 'ards':ti,ab,kw OR cardiovascular:ti,ab,kw OR cerebrovascular:ti,ab,kw OR liver:ti,ab,kw OR kidney:ti,ab,kw OR brain:ti,ab,kw OR heart:ti,ab,kw OR stroke:ti,ab,kw OR obesity:ti,ab,kw

#4 #1 AND #2 AND #3

#5 Filter applied: observational study, cohort analysis, case control study, case report, cross sectional study

Science Citation Index Expanded (Web of Science)

#1 TS= ('2019 ncov' OR 'severe acute respiratory syndrome coronavirus 2' OR 'covid 19' OR 'coronavirus disease 2019' OR 'novel coronavirus' OR 'new coronavirus' OR 'sars-cov-2')

#2 TS= (feature OR characteristic* OR prevalence OR symptom* OR predict* OR severity OR severe OR surviv* OR death OR die OR mortality OR mild OR moderate OR critical*)

#3 TS= (co-morbidit* OR comorbidit* OR complication OR chronic OR hypertension OR diabetes OR cancer OR tumor OR 'Chronic obstructive pulmonary disease' OR 'COPD' OR 'Acute respiratory distress syndrome' OR 'ARDS' OR cardiovascular OR cerebrovascular OR liver OR kidney OR brain OR heart OR stroke OR obesity)

#4 TS= (cohort OR case series OR case OR observational OR case-control OR cross-sectional)

#5 #1 AND #2 AND #3 AND #4

Cumulative Index to Nursing and Allied Health (CINAHL) Complete

#1 AB: '2019 ncov' OR 'severe acute respiratory syndrome coronavirus 2' OR 'covid 19' OR 'coronavirus disease 2019' OR 'novel coronavirus' OR 'new coronavirus' OR 'sars-cov-2'

#2 AB: feature OR characteristic* OR prevalence OR symptom* OR predict* OR severity OR severe OR surviv* OR death OR die OR mortality OR mild OR moderate OR critical*

#3 AB: co-morbidit* OR comorbidit* OR complication OR chronic OR hypertension OR diabetes OR cancer OR tumor OR 'Chronic obstructive pulmonary disease' OR 'COPD' OR 'Acute respiratory distress syndrome' OR 'ARDS' OR cardiovascular OR cerebrovascular OR liver OR kidney OR brain OR heart OR stroke OR obesity

#4 AB: cohort OR case series OR case OR observational OR case-control OR cross-sectional

#5 #1 AND #2 AND #3 AND #4

Appendix 2. Characteristics of the included studies

Supplemental Table 1. Characteristics of the included studies

ID	Country	Province/State	Type of study	Type of comparisons	Number of patients	Percentage of male	Age*	Multi-center study	Date of patients' admission※
Huang C	China	Hubei	case series	ICU vs non-ICU	41	73.17	49.0 (41.0-58.0)	No	December 16, 2019, to January 2, 2020
Liu J	China	Hubei	case series	severe vs non-severe	40	37.5	48.7±13.9	No	January 5 to January 24
Yuan M	China	Hubei	case series	death vs survival	27	44.44	60 (47–69)	No	January 1 to January 25
Yang X	China	Hubei	case series	death vs survival	52	67.31	59.7±13.3	No	December 24, 2019, to January 26, 2020
Wu C	China	Hubei	retrospective cohort study	ARDS vs non-ARDS; death vs survival	201; 84	63.68; 71.43	58.5 (50.0-69.0) 51 (43-60)	No	December 25, 2019, to January 26, 2020
Chen G	China	Hubei	case series	severe vs non-severe	21	80.95	56.0 (50.0-65.0)	No	late December 2019 to January 27, 2020
Wang D	China	Hubei	case series	ICU vs non-ICU	138	54.35	56 (42-68)	No	January 1 to January 28
Guan W	China	30 provinces	case series	severe vs non-severe; ICU vs non-ICU	1099	58.12	47.0 (35.0-58.0)	Yes	December 11, 2019 to January 29, 2020
#Chen R	China	31 provinces	retrospective cohort study	death vs survival	1590	57.29	48.9±15.7	Yes	November 21, 2019 to January 31, 2020
#Liang W	China	31 provinces	retrospective cohort study	ICU vs non-ICU	1590	57.29	48.9±15.7	Yes	November 21, 2019 to January 31, 2020
Zhou F	China	Hubei	retrospective cohort study	death vs survival	191	62.3	56.0 (46.0-67.0)	Yes	December 29, 2019 to February 1, 2020

Cao J	China	Hubei	case series	death vs survival	102	51.96	54 (37-67)	No	January 3 to February 1
Xu JQ	China	Sichuan	case series	death vs survival	239	59.83	62.5±13.3	Yes	January 12 to February 3
Zhang JJ	China	Hubei	case series	severe vs non-severe	140	50.71	57 (25-87)	No	January 16 to February 3
Liu F	China	Shanghai	retrospective cohort study	severe vs non-severe	134	47.01	51.5 (37.0-65.0)	No	January 20 to February 3
Zheng Ya	China	Hubei	case series	severe vs non-severe	73	54.79	43 (21-76)	No	January 16 to February 4
Zhang SX	China	Ningxia	case series	severe vs non-severe	34	58.82	41±17	No	January 22 to February 4
Lei S	China	Hubei	retrospective cohort study	ICU vs non-ICU	34	41.18	55 (43-63)	Yes	January 1 to February 5
Li X	China	Hubei	ambispective cohort study	severe vs non-severe	548	50.91	60 (48-69)	No	January 26 to February 5
Wang L	China	Hubei	case series	death vs survival	339	48.97	69 (65-76)	No	January 1 to February 6
Zhang Ja	China	Hubei	retrospective cohort study	severe vs non-severe; death vs survival	663	48.42	55.6 (44-69)	No	January 11 to February 6
Du RHa	China	Hubei	prospective cohort study	death vs survival	179	54.19	57.6±13.7	No	December 25, 2019 and February 7, 2020
Ye CY	China	Zhejiang	case series	severe vs non-severe	856	51.29	46 (36-56)	Yes	January 17 to February 7
Zheng F	China	Hunan	case series	severe vs non-severe	161	49.69	45 (33.5-57)	No	January 17 to February 7
Zhou Y	China	Sichuan	case series	severe vs non-severe	366	56.56	43 (31.8-51.0)	Yes	January 20, 2019 to February 8, 2020
Wan S	China	Chongqing	case series	severe vs non-severe	135	54.07	47 (36-55)	No	January 23 to February 8
Chen TL	China	Hubei	case series	death vs survival	55	61.82	74 (65-91)	No	January 1 to February 10
Zhang R	China	Hubei	case series	severe vs non-severe	120	35.83	45.4±15.6	No	January 1 to February 10
Zhang G	China	Hubei	case series	severe vs non-severe	221	48.87	55.0 (39.0-66.5)	No	January 2 to February 10

Zou X	China	Hubei	retrospective cohort study	death vs survival	154	43.51	60.68 ±13.00	No	January 10 to February 10
Zhao X	China	Hubei	case series	severe vs non-severe	91	53.85	46.0▲	No	January 16 to February 10
Huang Q	China	Hunan	case series	severe vs non-severe	54	51.85	41 (31-51)	Yes	January 17 to February 10
Huang R	China	Jiangsu	case series	severe vs non-severe	202	57.43	44.0 (33.0-54.0)	Yes	January 22 to February 10
Wang F	China	Hubei	case series	ICU vs non-ICU	28	75.00	68.6±9.0	No	January 29 to February 10
Cen Y	China	Hubei	prospective cohort study	death vs survival	287	60.63	61(49-68)	Yes	up to February 10
Wang DW	China	Hubei	case series	death vs survival	107	53.27	51.0 (36.0-65.0)	Yes	up to February 10
Yao Q	China	Hubei	retrospective cohort study	severe vs non-severe; death vs survival	108; 25	39.81; 52	52 (37–58); 65(51–73.5); 56 (50.5–63.5)	No	January 30 to February 11
Chen T	China	Hubei	case series	death vs survival	274	62.41	62.0 (44.0-70.0)	No	January 13 to February 12
Zhang SY	China	Zhejiang	case series	severe vs non-severe	788	51.65	37.5-70▲	No	January 17 to February 12
Yang Qa	China	Hubei	case series	severe vs non-severe	136	48.53	56 (44-64)	No	January 28 to February 12
Zhang Y	China	Hubei	retrospective cohort study	severe vs non-severe	258	53.49	64 (56-70)	No	January 29 to February 12
Ren H	China	Hubei	case series	severe vs non-severe	151	51.66	59.5 ± 15.9	No	January 12 to February 13
He Fa	China	Hubei	case series	Severe vs non-severe	95	55.79	57.3 ± 14.7	No	January 17 to February 13
Du RHb	China	Hubei	case series	ICU vs non-ICU	109	70.48	70.7±10.9	Yes	December 25, 2019, to February 15, 2020
Feng Y	China	Hubei, Shanghai, Anhui	case series	severe vs non-severe	476	56.93	53 (40-64)	Yes	January 1 to February 15
Liu Da	China	Hubei	case series	death vs survival	599	54.26	72 66.5-82.0)	Yes	January 20 to February 15

Sun L	China	Beijing	case series	severe vs non-severe	55	56.36	44.0 (34.0-56.0)	No	January 20 to February 15
Hou H	China	Hubei	case series	severe vs non-severe	389	51.41	61.3±13.8	No	January 24 to February 15
Zhao S	China	Hubei	retrospective cohort study	death vs survival	83	69.88	64 ±11.0	No	January 31 to February 15
Zhang Jb	China	Hubei	case series	ICU vs non-ICU	111	41.44	38 (32-57)	No	January 13 to February 16
Hu XS	China	Hunan	retrospective cohort study	severe vs non-severe<br>ICU vs non-ICU	213	47.89	44 (34–58)	Yes	January 24 to February 16
Zhang N	China	Hubei, Hunan	case series	death vs survival	60	71.67	64.4 ± 11.0	Yes	January 9 to February 19
Mao L	China	Hubei	case series	severe vs non-severe	214	40.65	52.7±15.5	Yes	January 16 to February 19
Yi P	China	Zhejiang	retrospective cohort study	severe vs non-severe	100	63.00	54 (42-64)	No	January 19 to February 19
Wu J	China	Jiangsu, Anhui	case series	severe vs non-severe	280	53.93	43.12±19.02	Yes	January 20 to February 19
Li YK	China	Hubei	case series	severe vs non-severe<br>death vs survival	25	48.00	61 (51-69)	No	January 1 to February 20
Hu L	China	Hubei	case series	severe vs non-severe	323	51.39	6 1(23-91)	No	January 8 to February 20
Zheng Yb	China	Sichuan	case series	ICU vs non-ICU	99	51.52	49.4±18.45	No	January 16 to February 20
Lu YM	China	Shanghai	case series	severe vs non-severe	53	64.15	60.5 (41.3-67.5)	No	January 20 to February 20
Zhu Z	China	Zhejiang	case series	severe vs non-severe	127	64.57	50.90±15.26	No	January 23 to February 20
Xia XT	China	Hubei	case series	severe vs non-severe	63	52.38	64.55±14.88;<br>62.25±15.07	No	January 26 to February 20
Luo X	China	Hubei	case series	death vs survival	298	50.34	57 (40-69)	No	January 30 to February 20
Ma X	China	Henan	case series	severe vs non-severe<br>death vs survival	523	55.26	44 (32-54)	Yes	up to February 20
Deng Y	China	Hubei	case series	death vs survival	225	55.11	54 (37-67)	Yes	January 1 to February 21

Cai Q	China	Guangdong	case series	severe vs non-severe	383	47.78	61 (52–65); 44.5 (34–57)	No	January 11 to February 21
Cao YK	China	Hubei	case series	death vs survival	101	66.34	56.6±15.1	Yes	January 5 to February 22
Xie H	China	Hubei	case series	severe vs non-severe	79	55.7	60.0 (48.0-66.0)	No	February 2 to February 23
Liu SP	China	Hubei	case series	ICU vs non-ICU	255	53.33	64 (24-92)	No	February 1 to February 24
He XW	China	Hubei	case series	death vs survival	54	62.96	68.0 (59.8-74.3)	No	February 3 to February 24
Yan YL	China	Hubei	retrospective cohort study	death vs survival	193	59.07	64(49-73)	No	January 10 to February 24
Wang Y	China	Hubei	case series	death vs survival	344	52.03	64 (52-72)	No	January 25 to February 25
Wang J	China	Anhui, Chongqing, Heilongjiang, Shanxi, Sichuan, Fujian, Guangxi	case series	severe vs non-severe	558	51.25	47 (35-57)	Yes	January 28 to February 25
Li Y	China	Hubei	case series	ICU vs non-ICU	135	51.11	64 (48-72)	No	February 10 to February 26
Zhao X	China	Hubei	retrospective cohort study	death vs survival	532	46.24	64.7 ± 13.1; 48.2 ± 14.4	No	January 7 to February 28
Yu CZ	China	Hubei	case series	death vs survival	1464	50.27	64 (51-71)	No	January 14 to February 28
Shang W	China	Hubei	case series	severe vs non-severe	443	49.66	56 (43.25-66.75)	No	January 16 to February 28
Duan J	China	Chongqing	case series	severe vs non-severe	348	52.87	58±15 44±15	Yes	January 1 to February 29
Xie JF	China	Hubei, Guangdong, Jiangsu	case series	death vs survival	733	65.08	65(56-73)	Yes	January 1 to February 29

Yang Qb	China	Hubei	retrospective cohort study	death vs survival	226	50.00	67.82± 15.73<br>49.93± 15.35	No	January 1 to February 29
Wang AB	China	Beijing	case series	ARDS vs non-ARDS	130	58.46	46.5 (34-62)	No	January 20 to February 29
Li K	China	Hubei	case series	severe vs non-severe	83	53.01	45.5±12.3	No	January to February
Zou L	China	Hubei	case series	severe vs non-severe<br>death vs survival	121	54.55	65.0 (55.0-71.5)	No	January 16 to March 3
Wei X	China	Hubei	case series	severe vs non-severe	252	51.59	64.8±13.3	No	February 13 to March 3
Liu MY	China	Hubei	case series	death vs survival	665	47.82	57.4±14.8	Yes	January 1 to March 4
Zhao Y	China	Hubei	case series	death vs survival	539	47.31	58 (43-69)	No	January 13 to March 4
Lee JY	Korea	Daegu, Gyeongsangbuk-do	case series	death vs survival , ICU vs non-ICU	98	44.90	72 (68-79)	Yes	February 18 to March 4
Zhang YT	China	Guangdong	case series	severe vs non-severe	1350	48.67	44.1±17.9	Yes	up to March 4
Gu T	China	32 provinces	nested case–control study	death vs survival	275	62.91	66.4 ±14.5	Yes	December 18, 2019 to March 8 2020
Lei F	China	Hubei	retrospective cohort study	severe vs non-severe	5771	47.2	56 (43-65)	Yes	December 20, 2019 to March 8, 2020
Xiong SQ	China	Hubei	case series	severe vs non-severe	116	68.97	58.5(47-69)	No	January 20 to March 8
Jiang Y	China	Hubei	case series	death vs survival	281	50.89	70 (65–77)	No	January 30 to March 8
Liao YF	China	Hubei	retrospective cohort study	severe vs non-severe	148	50.00	55 (48-61)<br>56(48-62)	No	February 5 to March 9
He Fb	China	Guangzhou	retrospective cohort study	severe vs non-severe	288	45.49	48.5 (34.3-62)	No	January 15 to March 10

Colombi D	Italy	Piacenza	case series	ICU vs non-ICU	236	75	68 (66-70)	No	February 17 to March 10
Chen Q	China	Zhejiang	case series	severe vs non-severe	145	54.48	47.5±14.6	No	January 1 to March 11
Wei YP	China	Hubei	case series	severe vs non-severe	276	56.20	51 (41-58)	No	January 27 to March 11
Xu PP	China	Hubei, Zhejiang, Anhui, Shandong, Jiangsu, Liaoning, Guizhou, Shanghai	case series	death vs survival<br>ICU vs non-ICU	692	53.9	46.1±15.2	Yes	January 10 and March 13
Du H	China	Hubei	retrospective cohort study	severe vs non-severe	164	51.22	61.8±13.6	No	February 15 to March 14
Xie YJ	China	Hubei	case series	severe vs non-severe	62	43.55	66 (53.3-73.0)	No	February 15 to March 14
Liu SQ	China	Jiangsu	Retrospective cohort study	severe vs non-severe	625	52.64	44.44±17.9	Yes	January 10 to March 15
Gao C	China	Hubei	retrospective cohort study	severe vs non-severe<br>death vs survival	2877	51.09	64.24±11.2	No	February 5 to March 15
Wang Q	China	Beijing	case series	severe vs non-severe	105	53.33	45.0 (33.5-59.5)	No	January 12 to March 17
Allameh SF	Iran	Tehran	case series	death vs survival	396	66.16	56.9±15.7	No	February 20 to March 17
Sabri A	Iran	Tehran	retrospective cohort study	death vs survival;<br>ICU vs non-ICU	63	-	54.1±15.5	Yes	February 21 to March 17
Berenguer J	Spain	127 centers in Spain	retrospective cohort study	death vs survival	4035	61.02	70 (56-80)	Yes	March 8 to March 17

Xiao LS	China	Jiangxi, Hubei	retrospective cohort study	severe vs non-severe	442	50.23	-	Yes	January 1 to March 18
Pan F	China	Hubei	case-control study	death vs survival	124	68.55	68 (61-75)	No	January 27 to March 19
Giacomelli A	Italy	Milan	prospective cohort study	death vs survival	233	69.10	61 (50–72)	No	February 21 to March 19
Murillo-Zamora E	Mexico	nationwide	retrospective cohort study	ICU vs non-ICU	740	57.30	43.7±14.9	Yes	February 28 to March 20
Liu Db	China	Hubei	retrospective cohort study	severe vs non-severe	2044	48.92	62 (51-70)	Yes	January 27 to March 21
Ebinger JE	US	California	case series	severe vs non-severe; ICU vs non-ICU; ARDS vs non-ARDS	442	57.92	52.72±19.65	Yes	February 26 to March 21
Huang J	China	Hubei	retrospective cohort study	death vs survival	299	53.51	53.4±16.7	No	January 25 to March 24
Zamanian M	Iran	Kermanshah	retrospective cohort study	death vs survival	245	63.30	54.68±19.21	No	February 22 to March 24
Ciceri F	Italy	Milan	case series	death vs survival	410	72.93	65 (56–75)	No	February 25 to March 24
Hwang JM	South Korea	Daegu	retrospective cohort study	death vs survival	103	50.49	67.62± 5.32	Yes	February 1 to March 25
Rastad H	Iran	Alborz	retrospective cohort study	death vs survival	2957	53.74	54.8±16.9	Yes	February 20 to March 25
Lagi F	Italy	Tuscany	case series	ICU vs non-ICU	84	65.48	62 (51-72)	No	February 25 to March 26

Buckner FS	US	Washington	case series	severe vs non-severe	105	50.48	69 (23-97)	Yes	March 2 to March 26
Escalera-Antezana JP	Bolivia	-	case series	death vs survival	107	51.4	43.9±17.6	Yes	March 2 to March 29
Hong KS	South Korea	Daegu	case series	ICU vs non-ICU	98	38.78	55.4±17.1	No	up to March 29
Covino M	Italy	Rome	case series	death vs survival	69	53.62	84 (82-89)	No	March 1 to March 30
Li T	China	Hubei	case series	severe vs non-severe	312	59.94	69.2 ± 7.3	No	February 1 to March 31
Zhang SQ	China	Guizhou	case series	severe vs non-severe	134	51.49	33 (21.8-46.3)	No	February 15 to March 31
Ferroni E	Italy	Lombardy and Veneto Regions, Reggio Emilia province	retrospective cohort study	death vs survival	42,926	62.6	69 (57–79)	Yes	February 21 to March 31
Zhou W	China	Anhui	case series	severe vs non-severe	80	60	47 (35-56)	No	January to March
Dreher M	Germany	North Rhine-Westphalia	case series	ARDS vs non-ARDS	50	66	65 (58–76)	No	February to March
Rath D	German	Tübingen	case series	death vs survival	123	62.60	68±15	No	February to March
Zeng JH	China	Guangdong	case series	ICU vs non-ICU	416	47.60	64(59.5-68)<br>45(33-57)	No	January 11 to April 1
Romero-Sánchez CM	Spain	Albacete	case series	severe vs non-severe	841	56.24	66.42±14.96	Yes	March 1 to April 1
Lapthorne S	Ireland	Cork	retrospective cohort study	death vs survival	46	71.74	63 (51-77)	No	March 6 to April 2

Aggarwal S	US	Lowa	case series	severe vs non-severe	16	75	67 (38–95)	No	March 1 to April 4
Gupta S	US	65 hospitals across the US	retrospective cohort study	death vs survival	2,215	64.83	60.5±14.5	Yes	March 4 to April 4
Kalligeros M	US	Rhode Island	retrospective cohort study	ICU vs non-ICU	103	61.17	60 (50-72)	Yes	February 17 to April 5
Simonnet A	France	Lille	retrospective cohort study	ICU vs non-ICU	124	72.58	60(51-70)	No	February 27 to April 5
Argenzian MG	US	New York	case series	ICU vs non-ICU	850	60.12	64 (51-77)<br>62 (52-72)	No	March 11 to April 6
Al-Sabah S	Kuwait	Kuwait City	retrospective cohort study	ICU vs non-ICU	1158	81.61	54 (46.4-63.4)<br>39.3(30.7-50.4)	No	February 24 to April 7
Sanchez-Pina JM	Spain	Madrid	case series	death vs survival	39	58.97	64.7(36-88)	No	March 7 to April 7
Piano S	Italy	2 regions of Northern Italy	case series	ICU vs non-ICU	565	63.19	66±15	Yes	February 22 to April 8
Petrilli CM	US	New York	prospective cohort study	ICU vs non-ICU	2729	61.27	54 (38-66)	Yes	March 1 to April 8
Bhargava A	US	Michigan	case series	ICU vs non-ICU	197	52.28	60.6±16.2	No	March 8 to April 8
Brill SE	UK	London	retrospective cohort study	death vs survival	450	60.44	72 (56-83)	No	March 10 to April 8
Hajifathalian K	US	New York, Massachusetts	retrospective cohort study	death vs survival	664	62.5	64±17	Yes	March 4 to April 9

Iaccarino G	Italy	nationwide	cross-sectional study	death vs survival	1591	64.00	66.5 (0.4△)	Yes	March 9 to April 9
Fumagalli C	Italy	Brescia, Florence	retrospective cohort study	death vs survival	516	66.86	67±13	Yes	February 22 to April 10
Lodigiani C	Italy	Lombardy	retrospective cohort study	ICU vs non-ICU	388	68.04	66 (55-75)	No	February 23 to April 10
Okoh AK	US	New Jersey	retrospective cohort study	death vs survival	251	51.39	62 (49–74)	No	March 10 to April 10
Rivera-Izquierdo M	Spain	Granada	case series	death vs survival	238	55.04	64.7 (24-97)	No	March 16 to April 10
Ortiz-Brizuela E	Mexico	Mexico	prospective cohort study	ICU vs non-ICU	140	60.71	49 (39-61)	No	February 26 to April 11
Ferguson J	US	California	case series	ICU vs non-ICU	72	52.78	60.4 (43.4-70.6)	Yes	March 13 to April 11
Vrillon A	France	Paris	prospective cohort study	death vs survival	76	44.74	90 (86-92)	No	March 14 to April 11
Deng M	China	Hubei	retrospective cohort study	severe vs non-severe	65	55.38	32.5 (30.5-37.5) 35.0 (29.0-37.0)	No	March 13 to April 12
Mendes A	Switzerland	Geneva	retrospective cohort study	death vs survival	235	43.40	86.3± 6.5	No	March 13 to April 14
Nikpouraghdam M	Iran	Tehran	case series	death vs survival	2964	65.96	55.50±15.15	No	February 19 to April 15
Labenz C	Germany	Rhineland-Palatinate	retrospective cohort study	ICU vs non-ICU	42	69.05	67.5 (54.5-75.25)	No	March 3 to April 15

Goodall JW	UK	London	retrospective cohort study	death vs survival	981	64.32	69 (56-80)	No	March 12 to April 15
Monteiro AC	US	California	retrospective cohort study	ICU vs non-ICU	112	66.07	61(45-74)	Yes	March 12 to April 16
Omrani AS	Qatar	Qatar	retrospective cohort study	ICU vs non-ICU	1409	82.82	39 (30-50)	Yes	February 28 to April 17
Genet B	France	Paris	retrospective cohort study	death vs survival	201	32.84	86.3 ±8.0	No	March 17 to April 18
Docherty AB	UK	England, Scotland, Wales	prospective cohort study	death vs survival	13364	59.71	72.9 (58.0-82.0)	Yes	January 31 to April 19
Borobia AM	Spain	Madrid	retrospective cohort study	death vs survival	2226	48.25	61 (46–78)	No	February 25 to April 19
Karagiannidis C	Germany	920 hospitals: Administrative claims data from the German Local Health Care Funds	case series	ICU vs non-ICU	10021	51.88	68.3±17.3	Yes	February 26 to April 19
Jourdes A	France	Toulouse	case series	ICU vs non-ICU	263	58.94	65 (54-76)	No	March 7 to April 20
Grasselli G	Italy	Lombardy	retrospective cohort study	death vs survival	3988	79.94	63 (55-69)	No	February 20 to April 22
Smith AA	US	iConnecticut, Massachusetts	retrospective cohort study	death vs survival	346	56.07	66.86	Yes	March 1 to April 22

Wendel Garcia PD	Europe	54 collaborating centers in 10 countries	prospective cohort study	death vs survival	398	75.38	71 (62-78)<br>62 (54-70)	Yes	March 13 to April 22
Polverino F	Italy	nationwide	retrospective cohort study	death vs survival	3179	68.29	69 (57-78)	Yes	March 25 to April 22
Bartoletti M	Italy	Bologna, Pesaro, Ancona, MilaNo, Modena, Piacenza, Rimini, Ravenna	retrospective cohort study	ARDS vs non-ARDS	1113	63.25	65.7±15.2	Yes	February 22 to April 23
Carrillo-Vega MF	Mexico	Epidemiological Surveillance System for Viral Respiratory Diseases	cross-sectional study	death vs survival	9946	57.84	48.15±14.35	Yes	February 27 to April 23
Bahl A	US	Metro Detroit catchment area	retrospective cohort study	death vs survival	1461	52.70	62 (50.0-74.0)	Yes	March 1 to April 23
Israelsen SB	Denmark	Copenhagen	case series	ICU vs non-ICU	175	48.57	71 (55-81)	No	March 10 to April 23
Khamis F	Oman	Muscat	case series	ICU vs non-ICU	63	84.13	48±16	Yes	February 24 to April 24
Tambe MP	India	Maharashtra	case series	death vs survival	197	89	45.8±17.3	No	March 31 to April 24
Bellan M	Italy	Novara, Alessandria, Vercelli	case series	death vs survival	407	58.72	71(58-80)	Yes	March 1 to April 28

Li G	China, European regions, and North America	multi-country dataset	case series	death vs survival	598	43.48	46-71▲	Yes	January 1 to April 30
Boari GEM	Italy	Brescia	retrospective cohort study	death vs survival	258	67.05	71.0±13.8	No	February 28 to April 30
Chinnadurai R	UK	Bury	retrospective cohort study	death vs survival	215	61.86	74(60-82)	No	March 23 to April 30
Kim SR	Korea	nationwide	retrospective cohort study	ICU vs non-ICU	2959	39.84	-	No	up to April 30
Russo V	Italy	Milan, Naples, Bergamo, Turin	case series	ARDS vs non-ARDS	192	59.9	67.7±15.2	Yes	February to April
El-Solh AA	US	New York	case series	death vs survival	1634	95.04	68.8±13.4	Yes	January 1 to May 1
Trecarichi EM	Italy	Catanzaro	retrospective cohort study	death vs survival	48	54.17	85± 8<br>78± 13	No	March 27 to May 6
Gupta N	India	New Delhi	case series	ICU vs non-ICU<br>death vs survival	200	58	40.03±17.03	No	March 20 to May 8
Caliskan T	Turkey	Istanbul	case-control study	ICU vs non-ICU<br>death vs survival	565	-	48.0±19.7	No	March 15 and May 10
Attaway AA	US	Ohio and Florida	retrospective cohort study	ICU vs non-ICU	160	50.63	72.5±1.7	Yes	March 8 to May 13
Ioannou GN	US	nationwide	retrospective cohort study	ICU vs non-ICU<br>death vs survival	10131	91.02	63.6±16.2	No	Feburary 28 to May 14
Yun K	South Korea	nationwide	case series	ICU vs non-ICU	7363	42.03	-	No	January 20 to May 15
Klang E	US	New York	case series	death vs survival	3406	57.57	-	Yes	March 1 to May 17

Cattelan AM	Italy	Veneto region	case series	ICU vs non-ICU	303	60.07	62(50-74)	No	February 22 to May 20
Alqahtani AM	Saudi Arabia	Riyadh	cross-sectional study	severe vs non-severe	458	86.90	-	No	March 1 to May 20
Di Castelnuovo A	Italy	nationwide	retrospective cohort study	death vs survival	3894	61.71	men: 67▲	Yes	February 19 to May 23
Alshukry A	Kuwait	Kuwait City	case series	severe vs non-severe death vs survival ICU vs non-ICU	253	69.57	non-severe: 44.67 ± 15.75; ICU survivors:46.36 ± 13.7; ICU death46.36 ± 13.7	No	February 24 to May 24
Rokni M	Iran	Zahedan	cross-sectional study	death vs survival	233	63.95	49.8	No	February 29 to May 24
Harrison SL	US	24 healthcare organizations	retrospective cohort study	death vs survival	31461	45.47	50 (35–63)	Yes	January 20 to May 26
Jimenez E	Spain	Madrid	case series	ICU vs non-ICU, death vs survival	1393	57.50	death:82(71.5-97); survival: 65(53-78)	No	March 1 to May 28
Islam MZ	Bangladesh	Dhaka	retrospective cohort study	death vs survival	1016	64.07	37.0 (28−49)	No	May1 to May 30
Huh K	South Korea	National Health Insurance Service database	case-control study	Severe vs non-severe	2231	39.04	-	Yes	up to May 30
Shah C	US	New Jersey	retrospective cohort study	death vs survival	487	56.06	78.4±11.5 64.1±18.5	No	January 1 to May 31

Hwang J	South Korea	Daegu	retrospective cohort study	death vs survival	340	38.24	79.08±7.81<br>74.76±6.70	Yes	February 17 to May 31
Rosenthal N	US	nationwide	retrospective cohort study	death vs survival	64781	49.35	56.1±19.9	Yes	April 1 to May 31
Cocconcelli E	Italy	Padova	case series	ICU vs non-ICU	102	73.53	68 (22-94)	No	March to May
Kokoszka-Bargiel I	Poland	Silesian	cross-sectional study	ICU vs non-ICU	53	66.04	72.2±12.3<br>62.4±10.4	No	March 10 to June 10
Abohamr SI	Saudi Arabia	Riyadh	case series	ICU vs non-ICU; death vs survival	768	76.69	46.36±13.7	No	March 23 to June 15
Bepouka BI	Congo	Kinshasa	retrospective cohort study	death vs survival	141	67.38	49.6±16.5	No	March 23 to June 15
Rodilla E	Spain	nationwide	case series	death vs survival	12226	57.40	67.5±16.1	Yes	March 1 to June 24
Yan XQ	China	Hunan	case series	severe vs non-severe	218	55.96	42.9 (32.0-52.3)	Yes	January 21 to June 27
Hernández-Galdamez DR	Mexico	Epidemiological Surveillance System for Viral Respiratory Diseases	cross-sectional study	ICU vs non-ICU	211,003	54.71	45.7±16.3	Yes	up to June 27
Kayina CA	India	New Delhi	case series	death vs survival	235	68.09	50.7±15.1	No	May 11 to June 28
Cortés-Tellés A	Mexico	Yucatan	ambispective cohort study	death vs survival	200	69.00	55 (41-65)	No	March 28 to June 30

de Andrade CLT	Brazil	Unified Health System for each state and federal district	cross-sectional study	death vs survival	89405	56.51	58.9±16.8	Yes	late February to June
Popov GT	Bulgaria	Sofia	case series	severe vs non-severe	138	63.04	52.9±16.4	No	March to June
Macedo MCF	Brazil	Bahia	retrospective cohort study	death vs survival	3896	60.01	68.8±15.9<br>57.7±18.9	No	March 3 to July 29
Nachega JB	Congo	Kinshasa	retrospective cohort study	severe vs non-severe	766	65.27	46 (34-58)	Yes	March 10 to July 31
de Souza CDF	Brazil	Alagoas	cross-sectional study	death vs survival	9807	47.54	70.21±8.37	Yes	February 26 to August 1
Gao J	China	Hubei, Zhejiang	case series	severe vs non-severe	139	51.80	60 (47-69)	Yes	Not mentioned (early COVID-19 patients)
Kunal S	India	Rajasthan	case series	death vs survival	108	64.81	51.2±17.7	No	-
Saleh A	Germany	Braunschweig, Bad Krozingen	prospective cohort study	ICU vs non-ICU	40	62.50	67±17	Yes	-
Renieris G	Greece	-	case series	death vs survival	74	72.97	-	Yes	from beginning of March
Smadja DM	France	Paris	prospective cohort study	ICU vs non-ICU	40	70	59.5 (54.25-70.5); 53 (37-65.4)	No	-

*mean ± standard deviation; median (interquartile range) was reported if mean ± standard deviation was not available; Age in severe and non-severe COVID-19 groups was reported respectively when age was not reported for all patients.
※Data of the table was sorted according to the latest time of patients' admission; #Data from the same study.
△Standard error
▲Median
*Data of the table was sorted according to the latest time of patients' admission; #Data from the same study.

Appendix 3. Quality of the included studies

Supplemental Table 2. Quality of the case series

ID	Were there clear criteria for inclusion in the case series?	Was the condition measured in a standard, reliable way for all the patients included in the case series?	Were valid methods used for identification of the condition for all the patients included in the case series?	Did the case series have consecutive inclusion of patients?	Did the case series have complete inclusion of patients?	Was there clear reporting of the demographics of the patients in the study?	Was there clear reporting of clinical information of the patients?	Were the outcomes or follow up results of cases clearly reported?	Was there clear reporting of the presenting site(s)/clinic(s) demographic information?	Was statistical analysis appropriate?	Score
Abohamr SI	1	1	1	1	0	1	1	1	1	1	9
Aggarwal S	1	1	1	1	0	1	1	1	1	1	9
Allameh SF	1	1	1	1	1	1	1	1	1	1	10
Alshukry A	1	1	1	1	1	1	1	1	1	1	10
Argenzian MG	1	1	1	1	1	1	1	1	1	1	10
Bellan M	1	1	1	1	1	1	1	1	1	1	10
Bhargava A	1	1	1	1	1	1	1	1	1	1	10
Buckner FS	1	1	1	1	1	1	1	1	1	1	10
Cai Q	1	1	1	1	1	1	1	1	1	1	10
Cao J	1	1	1	1	1	1	1	1	1	1	10
Cao YK	1	1	1	1	0	1	1	1	1	1	9
Cattelan AM	1	1	1	0	0	1	1	1	1	1	8
Chen G	1	1	1	0	1	1	1	1	1	1	9
Chen Q	1	1	1	1	1	1	1	1	1	1	10
Chen T	1	1	1	1	1	1	1	1	1	1	10
Chen TL	1	1	1	0	1	1	1	1	1	1	9
Ciceri F	1	1	1	1	1	1	1	1	1	1	10
Cocconcelli E	1	1	1	1	0	1	1	1	1	1	9
Colombi D	1	1	1	1	0	1	1	1	1	1	9
Covio M	1	1	1	1	0	1	1	1	1	1	9
Deng Y	1	1	1	0	1	1	1	1	1	1	9
Dreher M	1	1	1	1	1	1	1	1	1	1	10
Du RHb	1	1	1	1	0	1	1	1	1	1	9
Duan J	1	1	1	0	0	1	1	1	1	1	8
Ebinger JE	1	1	1	1	1	1	1	1	1	1	10
El-Solh AA	1	1	1	1	1	1	1	1	0	1	9
Escalera-Anteza1 JP	1	1	1	1	1	1	1	1	0	1	9
Feng Y	1	1	1	0	1	1	1	1	1	1	9

Ferguson J	1	1	1	1	0	1	1	1	1	1	9
Gao J	1	1	1	1	1	1	1	1	1	1	10
Guan W	1	1	1	1	1	1	1	1	0	1	9
Gupta N	1	1	1	1	1	1	1	1	1	1	10
He Fa	1	1	1	1	1	1	1	1	1	1	10
He XW	1	1	1	0	0	1	1	1	1	1	8
Hong KS	1	1	1	1	1	1	1	1	1	1	10
Hou H	1	1	1	1	1	1	1	1	1	1	10
Hu L	1	1	1	1	0	1	1	1	1	1	9
Huang C	1	1	1	0	1	1	1	1	1	1	9
Huang Q	1	1	1	0	1	1	1	1	1	1	9
Huang R	1	1	1	1	1	1	1	1	1	1	10
Israelsen SB	1	1	1	1	1	1	1	1	1	1	10
Jiang Y	1	1	1	0	1	1	1	1	1	1	9
Jimenez E	1	1	1	1	0	1	1	1	1	1	9
Jourdes A	1	1	1	1	0	1	1	1	1	1	9
Karagiannidis C	1	1	1	1	1	1	1	1	1	1	10
Kayi1 CA	1	1	1	1	1	1	1	1	1	1	10
Khamis F	1	1	1	0	1	1	1	1	0	1	8
Klang E	1	1	1	1	0	1	1	1	1	1	9
Ku1l S	1	1	1	1	0	1	1	1	1	1	9
Lagi F	1	1	1	0	1	1	1	1	1	1	9
Lee JY	1	1	1	0	1	1	1	1	0	1	8
Li G	1	1	1	0	0	1	1	1	0	1	7
Li K	1	1	1	0	1	1	1	1	1	1	9
Li T	1	1	1	0	1	1	1	1	1	1	9
Li Y	1	1	1	1	1	1	1	1	1	1	10
Li YK	1	1	1	1	1	1	1	1	1	1	10
Liu Da	1	1	1	0	0	1	1	1	0	1	7
Liu J	1	1	1	0	1	1	1	1	1	1	9
Liu MY	1	1	1	1	0	1	1	1	1	1	9
Liu SP	1	1	1	1	1	1	1	1	1	1	10
Lu YM	1	1	1	0	0	1	1	1	1	1	8
Luo X	1	1	1	1	0	1	1	1	1	1	9
Ma X	1	1	1	0	0	1	1	1	0	1	7
Mao L	1	1	1	1	1	1	1	1	1	1	10
Nikpouraghdam M	1	1	1	1	1	1	1	1	1	1	10
Piano S	1	1	1	0	0	1	1	1	0	1	7
Popov GT	1	1	1	1	0	1	1	1	1	1	9
Rath D	1	1	1	1	1	1	1	1	1	1	10

Ren H	1	1	1	0	1	1	1	1	1	1	9
Renieris G	1	1	1	0	0	0	1	1	0	1	6
Rivera-Izquierdo M	1	1	1	0	1	1	1	1	1	1	9
Rodilla E	1	1	1	1	0	1	1	1	0	1	8
Romero-Sánchez CM	1	1	1	1	1	1	1	1	1	1	10
Russo V	1	1	1	1	1	1	1	1	1	1	10
Sanchez-Pi1 JM	1	1	1	1	1	1	1	1	1	1	10
Shang W	1	1	1	0	1	1	1	1	1	1	9
Sun L	1	1	1	0	1	1	1	1	1	1	9
Tambe MP	1	1	1	1	1	1	1	1	1	1	10
Wan S	1	1	1	0	1	1	1	1	1	1	9
Wang AB	1	1	1	0	0	1	1	1	1	1	8
Wang D	1	1	1	1	1	1	1	1	1	1	10
Wang DW	1	1	1	1	1	1	1	1	1	1	10
Wang F	1	1	1	0	1	1	1	1	1	1	9
Wang J	1	1	1	0	1	1	1	1	0	1	8
Wang Lb	1	1	1	1	0	1	1	1	1	1	9
Wang Q	1	1	1	0	0	1	1	1	1	1	8
Wang Y	1	1	1	0	0	1	1	1	1	1	8
Wei X	1	1	1	1	1	1	1	1	1	1	10
Wei YP	1	1	1	1	1	1	1	1	1	1	10
Wu J	1	1	1	0	1	1	1	1	1	1	9
Xia XT	1	1	1	0	0	1	1	1	1	1	8
Xie H	1	1	1	1	0	1	1	1	1	1	9
Xie JF	1	1	1	1	1	1	1	1	0	1	9
Xie YJ	1	1	1	1	0	1	1	1	1	1	9
Xiong SQ	1	1	1	0	1	1	1	1	1	1	9
Xu JQ	1	1	1	0	1	1	1	1	1	1	9
Xu PP	1	1	1	1	0	1	1	1	1	1	9
Yan XQ	1	1	1	1	1	1	1	1	1	1	10
Yang Q	1	1	1	1	0	1	1	1	1	1	9
Yang X	1	1	1	0	0	1	1	1	1	1	8
Ye CY	1	1	1	1	1	1	1	1	0	1	9
Yu CZ	1	1	1	1	0	1	1	1	1	1	9
Yuan M	1	1	1	1	1	1	1	1	1	1	10
Yun K	1	1	1	0	0	1	1	1	1	1	8
Zeng JH	1	1	1	1	1	1	1	1	1	1	10
Zhang G	1	1	1	0	1	1	1	1	1	1	9
Zhang Jb	1	1	1	0	0	1	1	1	1	1	8
Zhang JJ	1	1	1	1	1	1	1	1	1	1	10

Zhang N	1	1	1	0	0	1	1	1	0	1	7
Zhang R	1	1	1	1	1	1	1	1	1	1	10
Zhang SQ	1	1	1	0	1	1	1	1	1	1	9
Zhang SX	1	1	1	0	1	1	1	1	1	1	9
Zhang SY	1	1	1	0	1	1	1	1	1	1	9
Zhang YT	1	1	1	1	1	1	1	1	0	1	9
Zhao XY	1	1	1	0	1	1	1	1	1	1	9
Zhao Y	1	1	1	0	1	1	1	1	1	1	9
Zheng F	1	1	1	1	1	1	1	1	1	1	10
Zheng Ya	1	1	1	1	1	1	1	1	1	0	9
Zheng Yb	1	1	1	1	1	1	1	1	1	1	10
Zhou W	1	1	1	0	1	1	1	1	1	1	9
Zhou Y	1	1	1	1	1	1	1	1	0	1	9
Zhu Z	1	1	1	1	1	1	1	1	1	1	10
Zou L	1	1	1	0	1	1	1	1	1	1	9

Supplemental Table 3. Quality of the cohort studies

ID	Were the two groups similar and recruited from the same population?	Were the exposures measured similarly to assign people to both exposed and unexposed groups?	Was the exposure measured in a valid and reliable way?	Were confounding factors identified?	Were strategies to deal with confounding factors stated?	Were the groups/participants free of the outcome at the start of the study (or at the moment of exposure) ?	Were the outcomes measured in a valid and reliable way?	Was the follow up time reported and sufficient to be long enough for outcomes to occur?	Was follow up complete, and if not, were the reasons for loss to follow up described and explored?	Were strategies to address incomplete follow up utilized?	Was appropriate statistical analysis used?	Score
Al-Sabah S	1	1	1	1	1	1	1	0	1	1	1	10
Attaway AA	1	1	1	1	1	1	1	0	1	1	1	10
Bahl A	1	1	1	1	1	1	1	1	1	1	1	11
Bartoletti M	0	1	1	1	1	1	1	0	1	1	1	9
Bepouka BI	1	1	1	1	1	1	1	1	1	1	1	11
Berenguer J	1	1	1	1	1	1	1	1	1	1	1	11
Boari GEM	1	1	1	1	1	1	1	1	1	1	1	11
Borobia AM	1	1	1	1	1	1	1	1	0	0	1	9
Brill SE	1	1	1	1	1	1	1	1	1	0	1	10
Cen Y	1	1	1	1	1	1	1	1	0	0	1	9
Chen R	0	1	1	1	1	1	1	1	1	1	1	10
Chinnadurai R	1	1	1	1	1	1	1	1	1	1	1	11
Cortés-Tellés A	1	1	1	1	1	1	1	1	1	1	1	11
Deng M	1	1	1	1	1	1	1	1	1	1	1	11
Di Castelnuovo A	0	1	1	1	1	1	1	1	1	1	1	10
Docherty AB	1	1	1	1	1	1	1	1	1	1	1	11
Du H	1	1	1	1	1	1	1	1	1	1	1	11
Du RH	1	1	1	1	1	1	1	1	1	1	1	11
Ferroni E	0	1	1	1	1	1	1	0	1	1	1	9
Fumagalli C	0	1	1	1	1	1	1	0	1	1	1	9
Gao C	1	1	1	1	1	1	1	1	1	1	1	11
Genet B	1	1	1	1	1	1	1	1	1	1	1	11
Giacomelli A	1	1	1	1	1	1	1	1	1	1	1	11

Goodall JW	1	1	1	1	1	1	1	0	1	1	1	10
Grasselli G	1	1	1	1	1	1	1	0	1	1	1	10
Gupta S	1	1	1	1	1	1	1	1	1	1	1	11
Hajifathalian K	0	1	1	1	1	1	1	1	1	1	1	10
Harrison SL	0	1	1	1	1	1	1	1	1	1	1	10
He Fb	1	1	1	1	1	1	1	0	1	1	1	10
Hu XS	0	1	1	1	1	1	1	1	1	1	1	10
Huang J	1	1	1	1	1	1	1	1	1	1	1	11
Hwang J	1	1	1	1	1	1	1	1	1	1	1	11
Hwang JM	1	1	1	1	1	1	1	0	1	1	1	10
Ioannou GN	1	1	1	1	1	1	1	1	1	1	1	11
Islam MZ	1	1	1	1	1	1	1	0	1	1	1	10
Kalligeros M	0	1	1	1	1	1	1	1	1	1	1	10
Kim SR	1	1	1	1	1	1	1	0	1	1	1	10
Labenz C	1	1	1	1	1	1	1	0	1	1	1	10
Lapthorne S	1	1	1	1	0	1	1	1	1	1	1	10
Lei F	0	1	1	1	1	1	1	1	1	1	1	10
Lei S	0	1	1	0	0	1	1	1	1	1	1	8
Li X	0	1	1	1	1	1	1	1	1	1	1	10
Liang W	0	1	1	1	1	1	1	1	1	1	1	10
Liao YF	1	1	1	1	1	1	1	1	0	1	1	10
Liu Db	0	1	1	1	1	1	1	0	1	1	1	9
Liu F	1	1	1	1	1	1	1	1	1	1	1	11
Liu SQ	0	1	1	1	1	1	1	1	1	1	1	10
Lodigiani C	1	1	1	1	1	1	1	0	1	1	1	10
Macedo MCF	1	1	1	1	1	1	1	0	1	1	1	10
Mendes A	1	1	1	1	1	1	1	0	1	1	1	10
Monteiro AC	0	1	1	1	1	1	1	1	1	0	1	9
Murillo-Zamora E	0	1	1	1	1	1	1	0	1	1	1	9
Nachega JB	0	1	1	1	1	1	1	0	1	1	1	9
Okoh AK	1	1	1	1	1	1	1	1	0	0	1	9
Omrani AS	0	1	1	1	1	1	1	1	1	1	1	10
Ortiz-Brizuela E	1	1	1	1	1	1	1	1	1	1	1	11
Petrilli CM	1	1	1	1	1	1	1	1	1	1	1	11

Polverino F	0	1	1	1	1	1	1	0	1	1	1	9
Rastad H	0	1	1	1	1	1	1	0	1	1	1	9
Rosenthal N	0	1	1	1	1	1	1	0	1	1	1	9
Sabri A	0	1	1	1	1	1	1	0	1	1	1	9
Saleh A	0	1	1	1	1	1	1	0	1	1	1	9
Shah C	1	1	1	1	1	1	1	0	1	1	1	10
Simonnet A	1	1	1	1	1	1	1	0	1	1	1	10
Smadja DM	1	1	1	1	1	1	1	0	1	1	1	10
Smith AA	0	1	1	0	0	1	1	0	1	1	0	6
Trecarichi EM	1	1	1	1	1	1	1	0	1	1	1	10
Vrillon A	1	1	1	1	1	1	1	1	1	1	1	11
Wendel Garcia PD	0	1	1	1	1	1	1	0	1	1	1	9
Wu C	1	1	1	1	1	1	1	1	1	1	1	11
Xiao LS	0	1	1	1	1	1	1	0	0	1	1	8
Yan YL	1	1	1	1	1	1	1	1	1	1	1	11
Yang Q	1	1	1	1	1	1	1	0	1	1	1	10
Yao Q	1	1	1	1	1	1	1	1	1	1	1	11
Yi P	1	1	1	1	1	1	1	0	1	1	1	10
Zamanian M	1	1	1	1	1	1	1	0	1	1	1	10
Zhang Ja	1	1	1	1	1	1	1	1	1	1	1	11
Zhang Y	1	1	1	1	1	1	1	0	1	1	1	10
Zhao S	1	1	1	1	1	1	1	1	1	1	1	11
Zhao X	1	1	1	1	1	1	1	1	0	0	1	9
Zhou F	1	1	1	1	1	1	1	1	1	1	1	11
Zhou X	1	1	1	1	1	1	1	1	1	1	1	11

Supplemental Table 4. Quality of the case-control studies

ID	Were the groups comparable other than the presence of disease in cases or the absence of disease in controls?	Were cases and controls matched appropriately?	Were the same criteria used for identification of cases and controls?	Was exposure measured in a standard, valid and reliable way?	Was exposure measured in the same way for cases and controls?	Were confoundi ng factors identified?	Were strategies to deal with confounding factors stated?	Were outcomes assessed in a standard, valid and reliable way for cases and controls?	Was the exposure period of interest long e0ugh to be meaningful?	Was appropriate statistical a1lysis used?	Score
Caliskan T	0	0	1	1	1	1	1	1	1	1	8
Gu T	1	1	1	1	1	1	1	1	1	1	10
Huh K	1	1	1	1	1	1	1	1	1	1	10
Pan F	0	0	1	1	1	1	1	1	1	1	8

Supplemental Table 5. Quality of the cross-sectional studies

ID	Were the criteria for inclusion in the sample clearly defined?	Were the study subjects and the setting described in detail?	Was the exposure measured(chronic disease) in a valid and reliable way?	Were objective, standard criteria used for measurement of the condition (COVID-19)?	Were confounding factors identified?	Were strategies to deal with confounding factors stated?	Were the outcomes measured in a valid and reliable way?	Was appropriate statistical analysis used?	score
Alqahtani AM	1	1	1	1	1	0	1	0	6
Carrillo-Vega MF	1	1	1	0	1	1	1	1	7
de Andrade CLT	1	1	1	1	1	1	1	1	8
de Souza CDF	1	1	1	0	1	1	1	1	7
Hernández-Galdamez DR	1	1	1	1	1	1	1	1	8
Iaccarino G	1	1	1	1	1	1	1	1	8
Kokoszka-Bargiel I	1	1	1	1	1	0	1	0	6
Rokni M	1	1	1	1	1	0	1	0	6

Appendix 4. Cumulative meta-analysis according to admission date and sample size

Supplemental Table 6. Hypertension for severity and mortality of COVID-19: cumulative meta-analysis

Type of comparisons	Season of admission	Pooled OR (95% CI)	Sample size	Number of the included studies	From which study the statistical test results of pooled OR became stable
severe vs non-severe	Winter	3.07 (2.45-3.85)	11901	39	3
	Spring	2.95 (2.27-3.83)	18009	25	8
	Summer	3.78 (2.73-5.22)	1165	2	1
death vs survival	Winter	2.70 (2.10-3.48)	11085	29	2
	Spring	2.28 (1.96-2.66)	193858	59	14
	Summer	1.70 (1.46-1.99)	123551	7	6
ICU vs non-ICU	Winter	2.65 (1.55-4.53)	4519	11	6
	Spring	2.25 (1.80-2.83)	62892	34	8
	Summer	1.66 (1.11-2.48)	254579	3	3

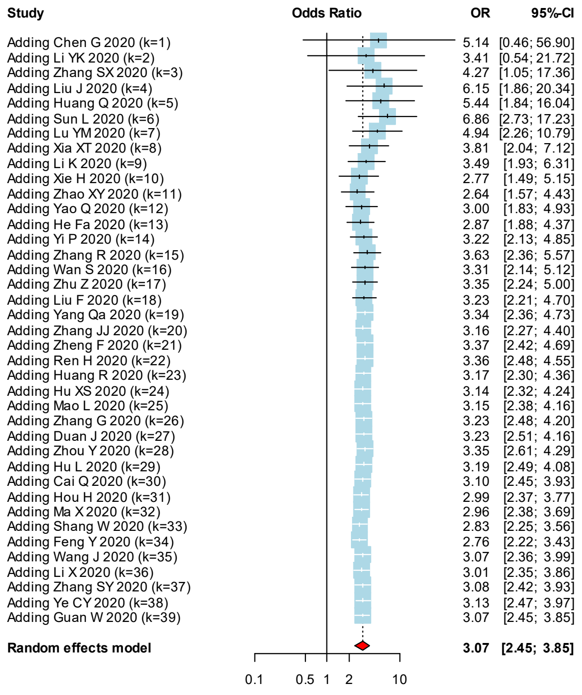

a: Patients' admission date in winter

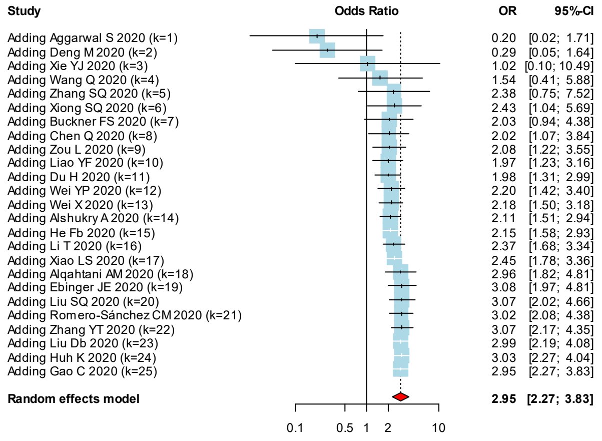

b: Patients' admission date in spring

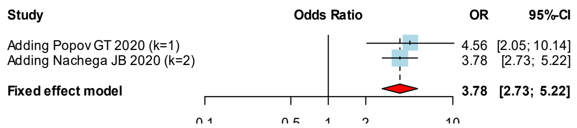

c: Patients' admission date in summer

Supplemental Figure 1: Association between hypertension and severity of COVID-19: cumulative meta-analysis

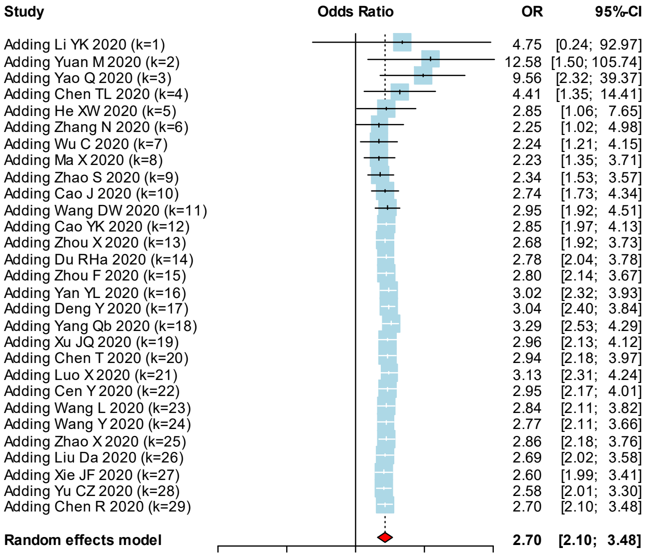

a: Patients' admission date in winter

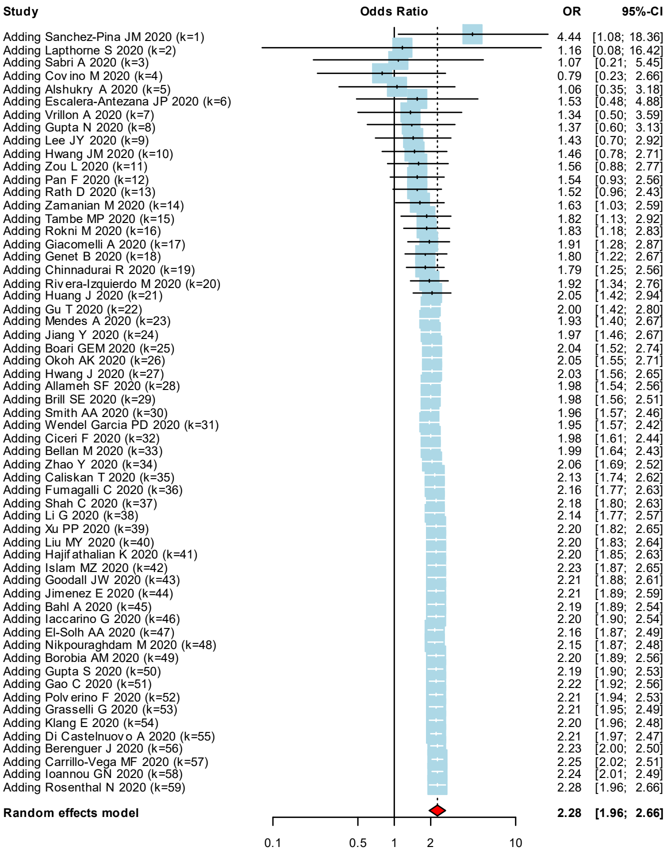

b: Patients' admission date in spring

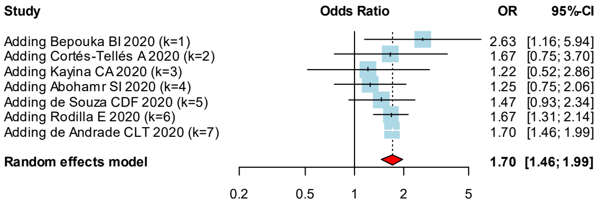

c: Patients' admission date in summer

Supplemental Figure 2: Association between hypertension and mortality of COVID-19: cumulative meta-analysis

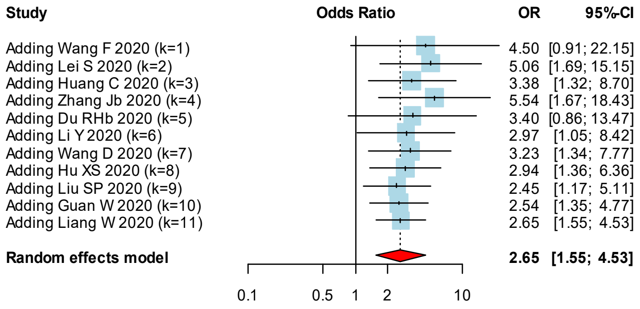

a: Patients' admission date in winter

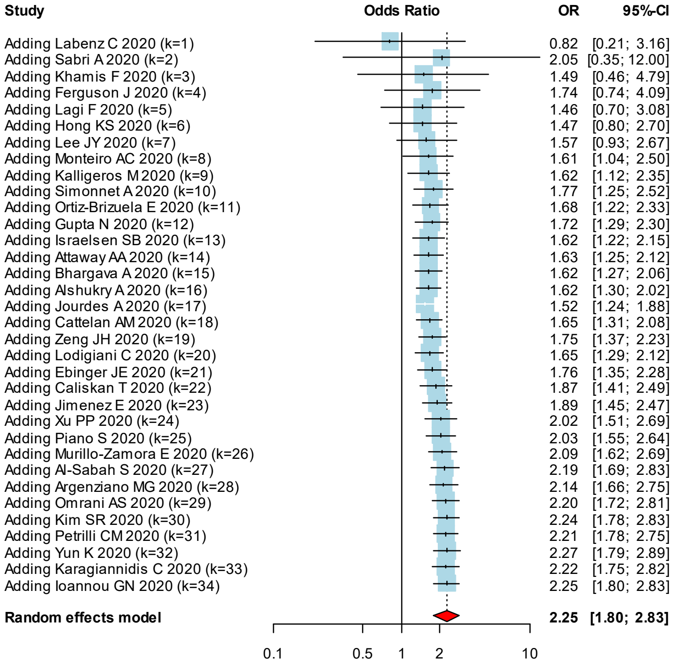

b: Patients' admission date in spring

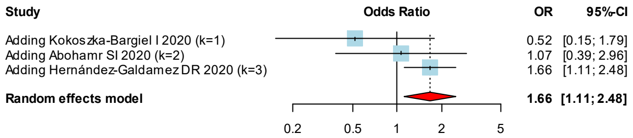

c: Patients' admission date in summer

Supplemental Figure 3: Association between hypertension and ICU admission of COVID-19: cumulative meta-analysis

Supplemental Table 7. Diabetes for severity and mortality of COVID-19: cumulative meta-analysis

Type of comparisons	Season of admission	Pooled OR (95% CI)	Sample size	Number of the included studies	From which study the statistical test results of pooled OR became stable
severe vs non-severe	Winter	2.21 (1.91-2.56)	11171	40	3
	Spring	2.48 (1.88-3.28)	19476	26	9
	Summer	4.46 (3.22-6.69)	1265	3	2
death vs survival	Winter	2.23 (1.75-2.85)	10023	30	7
	Spring	1.95 (1.74-2.19)	225248	65	9
	Summer	1.87 (1.52-2.30)	123151	8	5
ICU vs non-ICU	Winter	2.95 (1.80-4.85)	4203	11	7
	Spring	2.40 (2.00-2.89)	55616	35	3
	Summer	2.60 (2.46-2.75)	246892	3	2

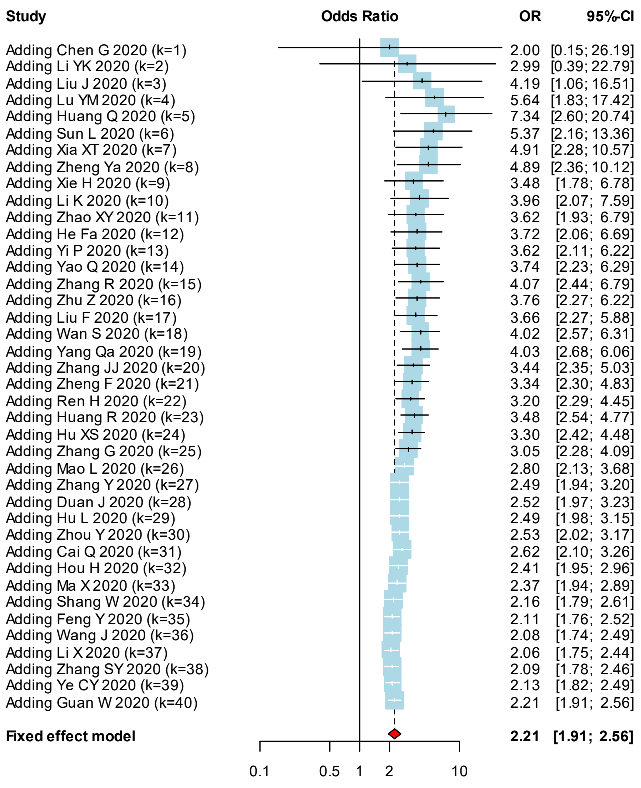

a: Patients' admission date in winter

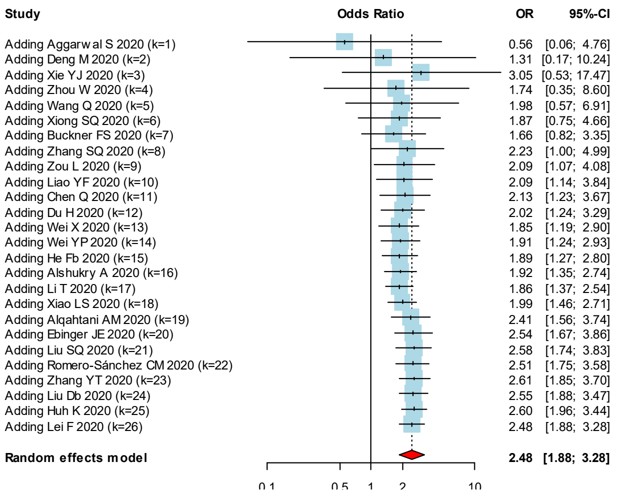

b: Patients' admission date in spring

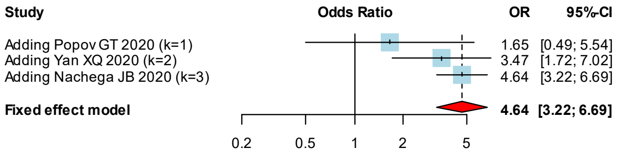

c: Patients' admission date in summer

Supplemental Figure 4: Association between diabetes and severity of COVID-19: cumulative meta-analysis

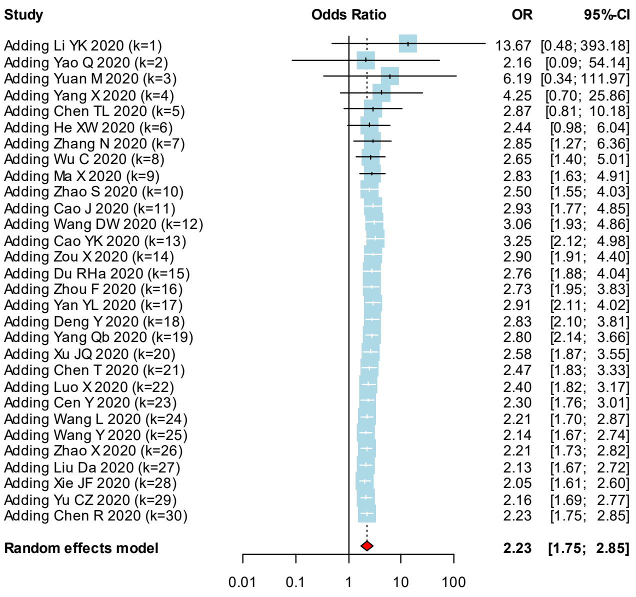

a: Patients' admission date in winter

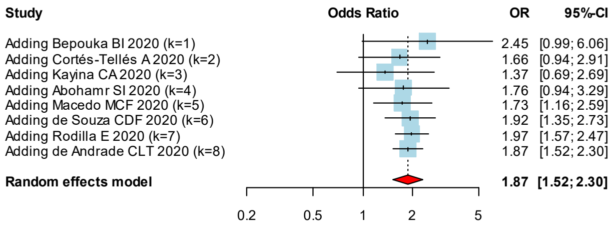

c: Patients' admission date in summer

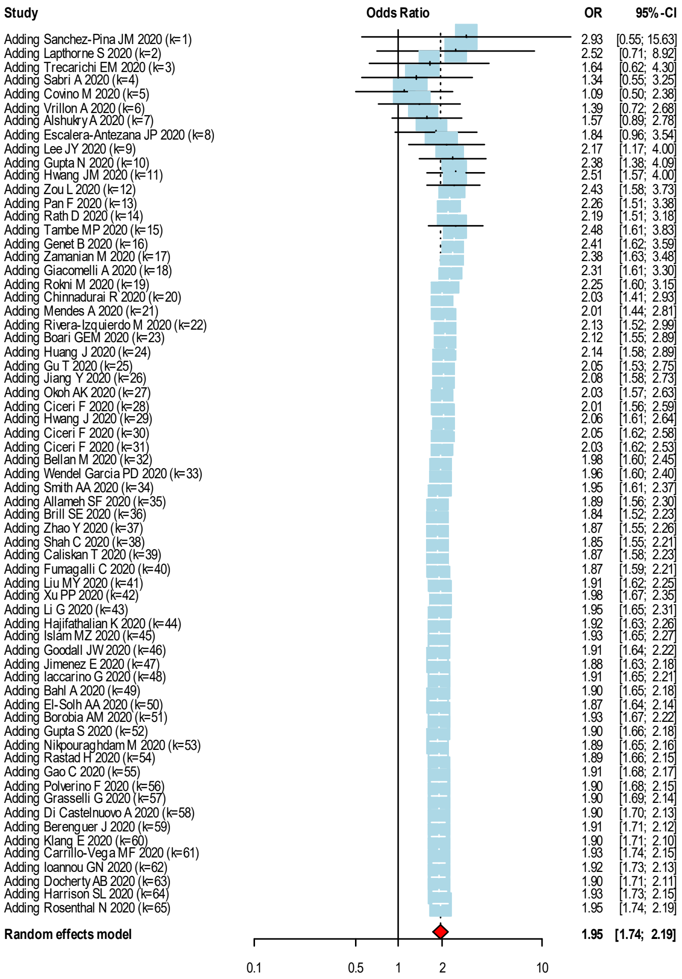

b: Patients' admission date in spring

Supplemental Figure 5: Association between diabetes and mortality of COVID-19: cumulative meta-analysis

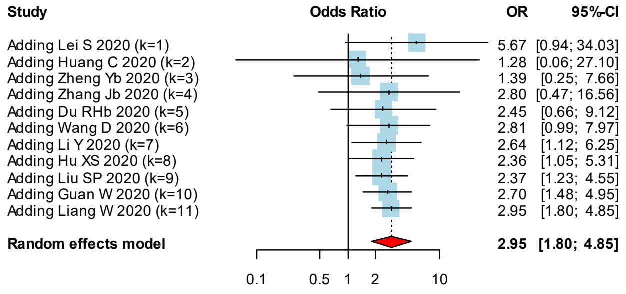

a: Patients' admission date in winter

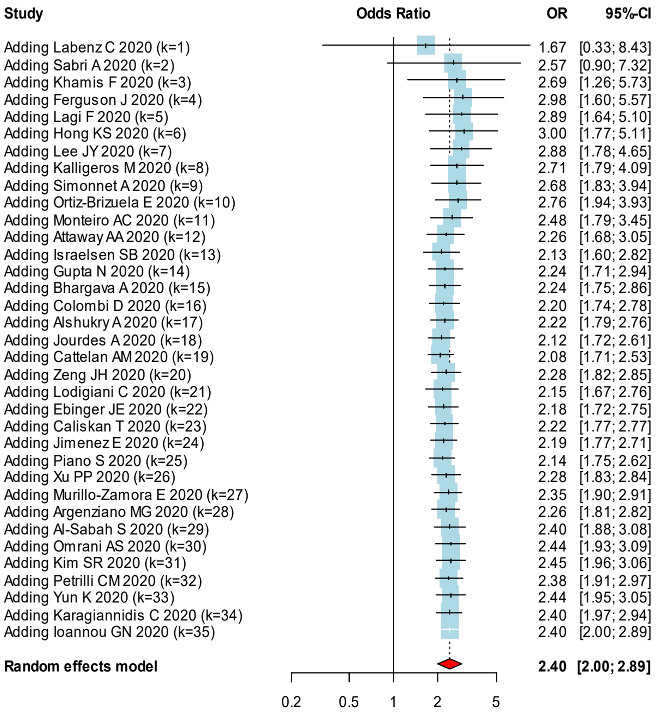

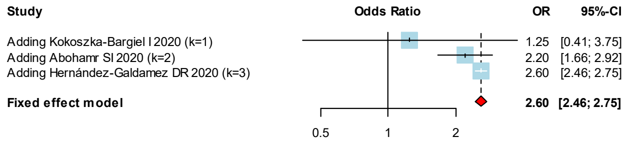

c: Patients' admission date in summer

b: Patients' admission date in spring

Supplemental Figure 6: Association between diabetes and ICU admission of COVID-19: cumulative meta-analysis

Supplemental Table 8. COPD for severity and mortality of COVID-19: cumulative meta-analysis

Type of comparisons	Season of admission	Pooled OR (95% CI)	Sample size	Number of the included studies	From which study the statistical test results of pooled OR became stable
severe vs non-severe	Winter	4.84 (3.55-6.61)	6675	19	1
	Spring	2.48 (1.59-3.87)	9864	12	8
death vs survival	Winter	4.75 (2.50-9.02)	3676	13	6
	Spring	2.80 (2.30-3.41)	54028	37	12
	Summer	2.44 (2.11-2.82)	13529	3	3
ICU vs non-ICU	Winter	7.61 (3.78-15.33)	1927	7	4
	Spring	2.40 (1.47-3.91)	20908	16	11
	Summer	2.11 (1.82-2.44)	214783	2	2

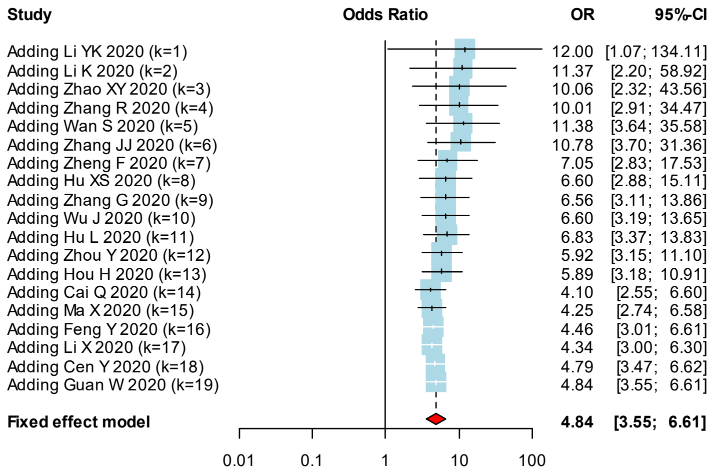

a: Patients' admission date in winter

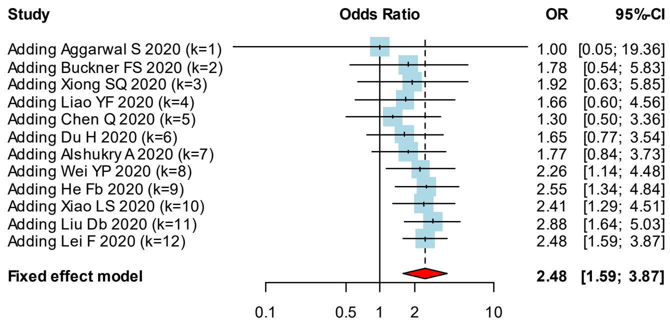

b: Patients' admission date in spring

Supplemental Figure 7: Association between COPD and severity of COVID-19: cumulative meta-analysis

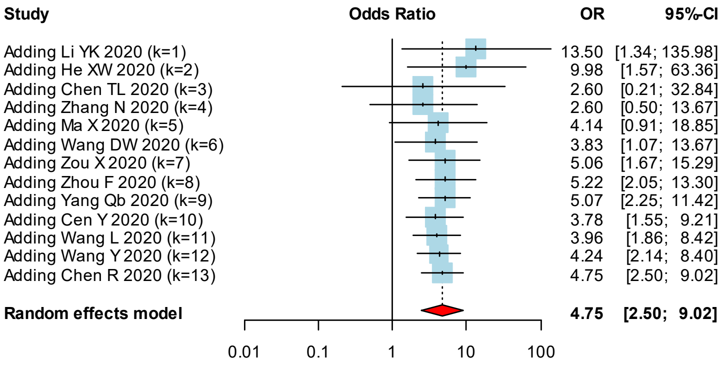

a: Patients' admission date in winter

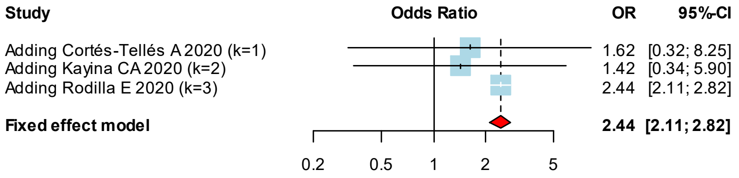

c: Patients' admission date in summer

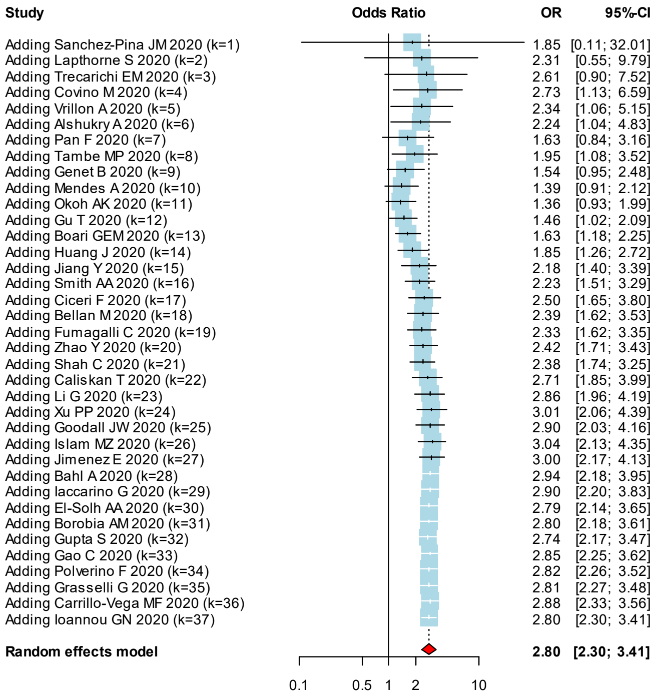

b: Patients' admission date in spring

Supplemental Figure 8: Association between COPD and mortality of COVID-19: cumulative meta-analysis

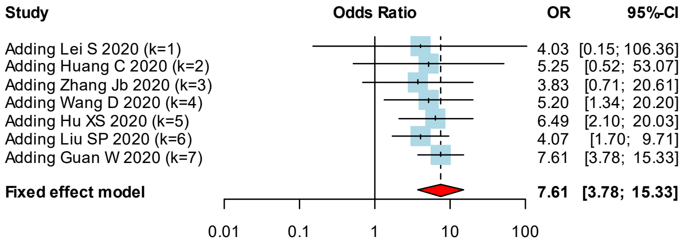

a: Patients' admission date in winter

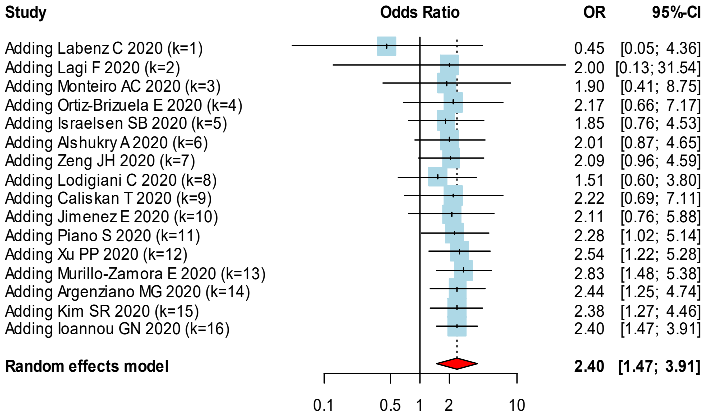

b: Patients' admission date in spring

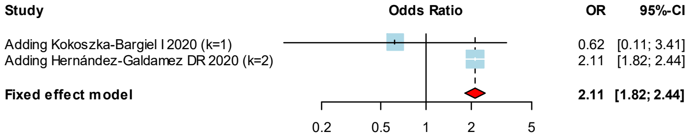

c: Patients' admission date in summer

Supplemental Figure 9: Association between COPD and ICU admission of COVID-19: cumulative meta-analysis

Supplemental Table 9. Coronary heart disease for severity and mortality of COVID-19: cumulative meta-analysis

Type of comparisons	Season of admission	Pooled OR (95% CI)	Sample size	Number of the included studies	From which study the statistical test results of pooled OR became stable
severe vs non-severe	Winter	2.92 (1.95-4.39)	3750	11	8
	Spring	2.63 (1.66-4.15)	9085	8	2
death vs survival	Winter	3.38 (2.05-5.58)	5515	11	3
	Spring	2.40 (2.06-2.80)	33497	21	6
	Summer	2.43 (2.12-2.79)	13450	2	2
ICU vs non-ICU	Winter	4.27 (1.30-14.07)	1675	4	4
	Spring	2.03 (1.45-2.84)	24109	14	11

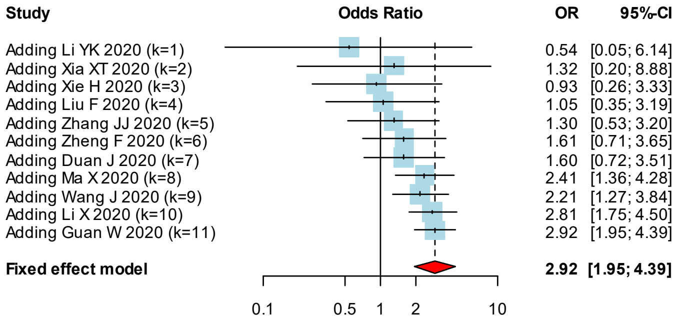

a: Patients’ admission date in winter

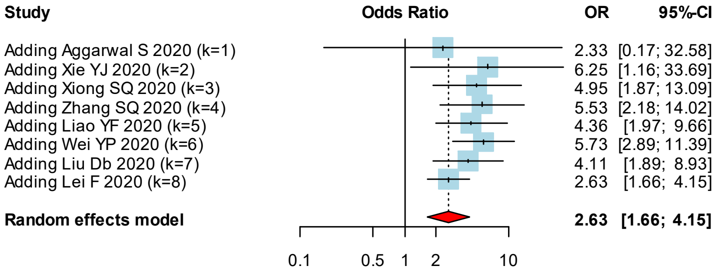

b: Patients’ admission date in spring

Supplemental Figure 10: Association between coronary heart disease and severity of COVID-19: cumulative meta-analysis

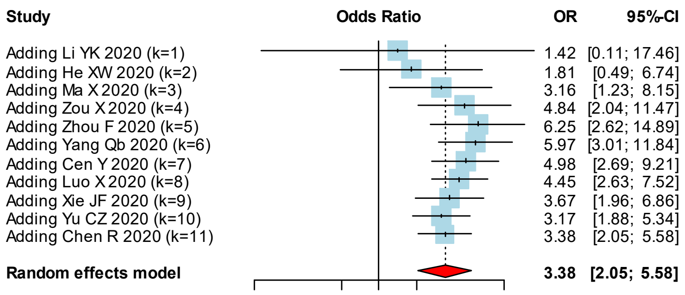

a: Patients’ admission date in winter

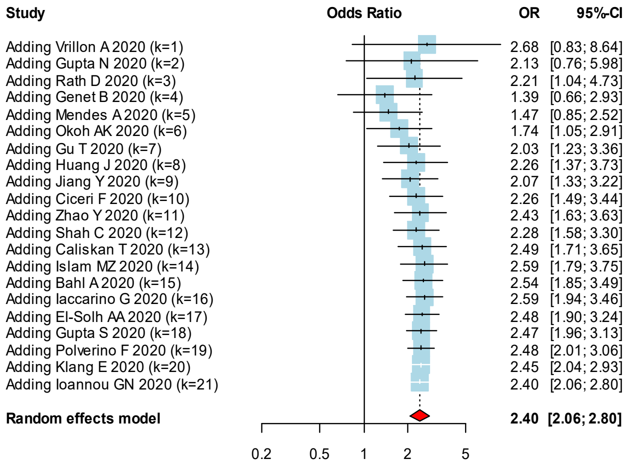

b: Patients’ admission date in spring

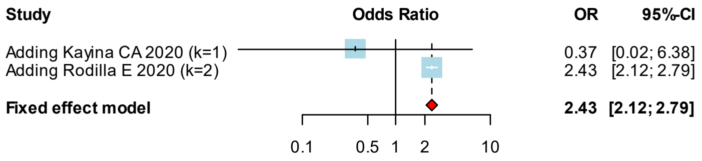

c: Patients’ admission date in summer

Supplemental Figure 11: Association between coronary heart disease and mortality of COVID-19: cumulative meta-analysis

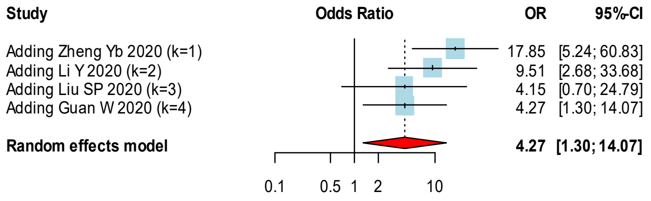

a: Patients' admission date in winter

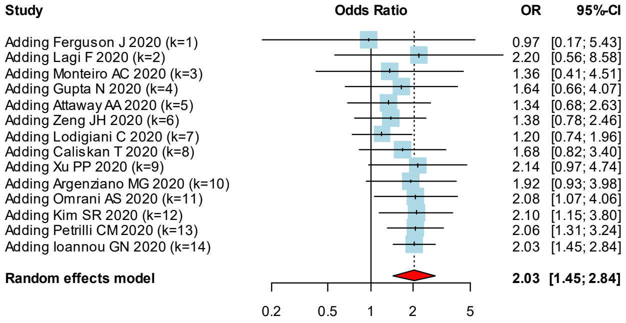

b: Patients' admission date in spring

Supplemental Figure 12: Association between coronary heart disease and ICU admission of COVID-19: cumulative meta-analysis

Supplemental Table 10. Cerebrovascular disease for severity and mortality of COVID-19: cumulative meta-analysis

Type of comparisons	Season of admission	Pooled OR (95% CI)	Sample size	Number of the included studies	From which study the statistical test results of pooled OR became stable
severe vs non-severe	Winter	3.37 (2.23-5.09)	4605	14	2
	Spring	1.81 (1.35-2.44)	8755	9	6
	Summer	7.92 (1.93-32.52)	365	2	2
death vs survival	Winter	2.33 (1.69-3.22)	6336	15	11
	Spring	2.53 (2.08-3.08)	126072	23	1
	Summer	1.31 (0.28-6.12)	13988	2	NA
ICU vs non-ICU	Winter	5.47 (3.21-9.32)	3309	7	3
	Spring	1.31 (0.68-2.54)	20572	9	NA
	Summer	0.41 (0.08-2.13)	880	2	NA

NA: not applicable due to non-statistically significance results.

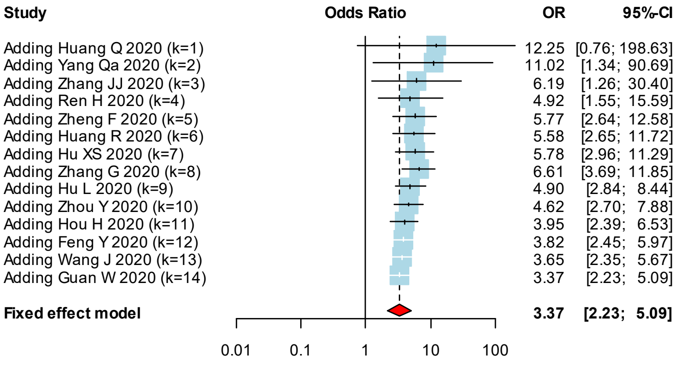

a: Patients' admission date in winter

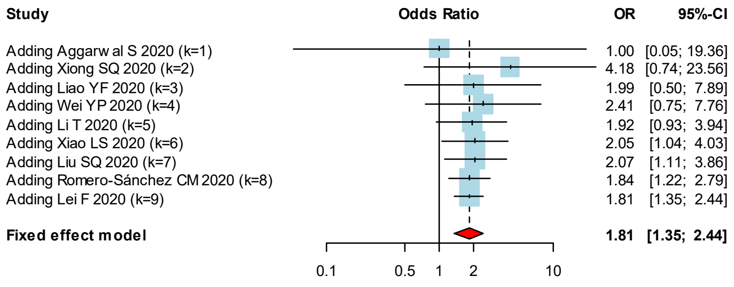

b: Patients' admission date in spring

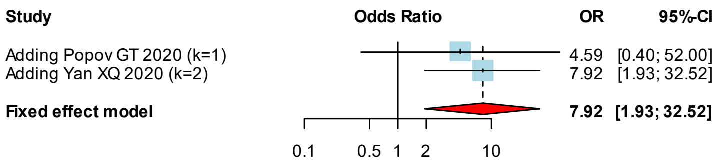

c: Patients' admission date in summer

Supplemental Figure 13: Association between cerebrovascular disease and severity of COVID-19: cumulative meta-analysis

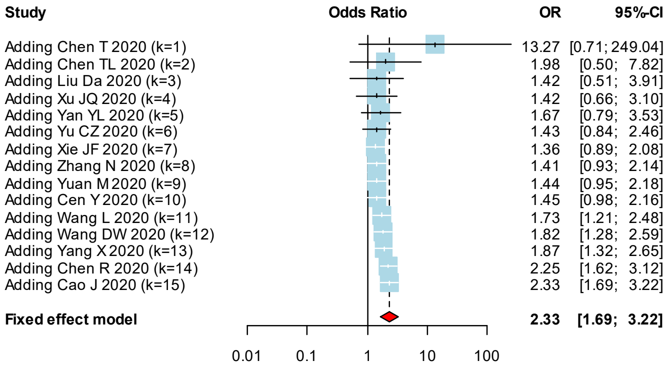

a: Patients' admission date in winter

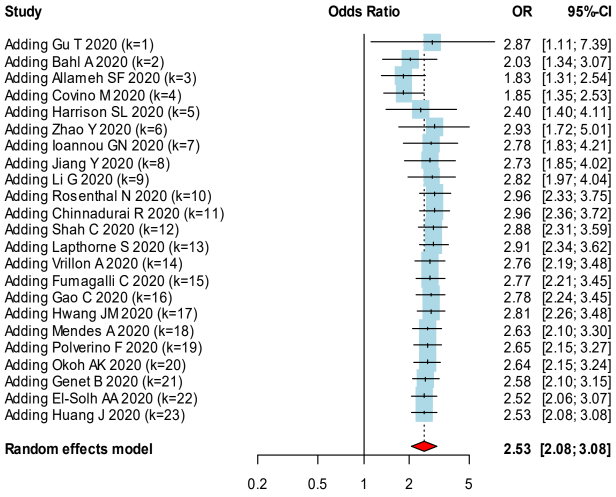

b: Patients' admission date in spring

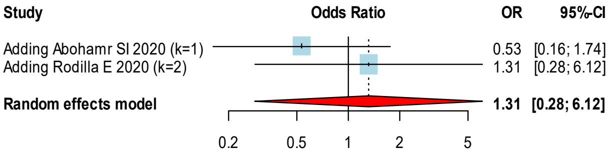

c: Patients' admission date in summer

Supplemental Figure 14: Association between cerebrovascular disease and mortality of COVID-19: cumulative meta-analysis

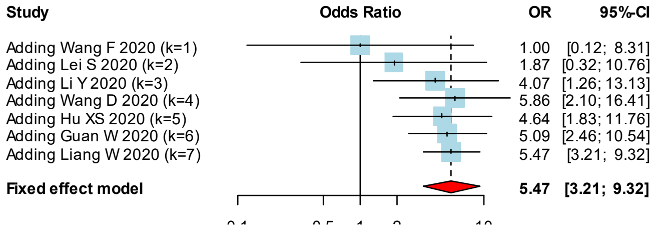

a: Patients' admission date in winter

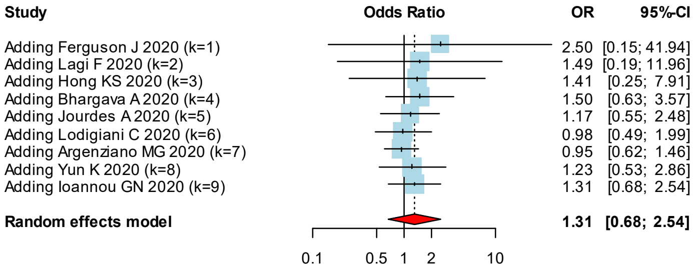

b: Patients' admission date in spring

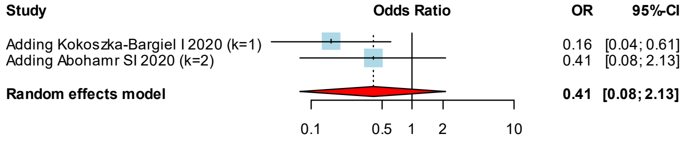

c: Patients' admission date in summer

Supplemental Figure 15: Association between cerebrovascular disease and ICU admission of COVID-19: cumulative meta-analysis

Supplemental Table 11. Cancer for severity and mortality of COVID-19: cumulative meta-analysis

Type of comparisons	Season of admission	Pooled OR (95% CI)	Sample size	Number of the included studies	From which study the statistical test results of pooled OR became stable
severe vs non-severe	Winter	2.79 (1.94-4.00)	8100	24	5
	Spring	2.03 (1.57-2.63)	5656	13	7
	Summer	5.57 (1.83-16.94)	1139	3	1
death vs survival	Winter	2.10 (1.42-3.09)	7184	18	16
	Spring	2.14 (1.85-2.47)	172823	46	14
ICU vs non-ICU	Winter	2.21 (1.24-3.93)	3403	8	8
	Spring	1.56 (1.38-1.76)	24821	20	10

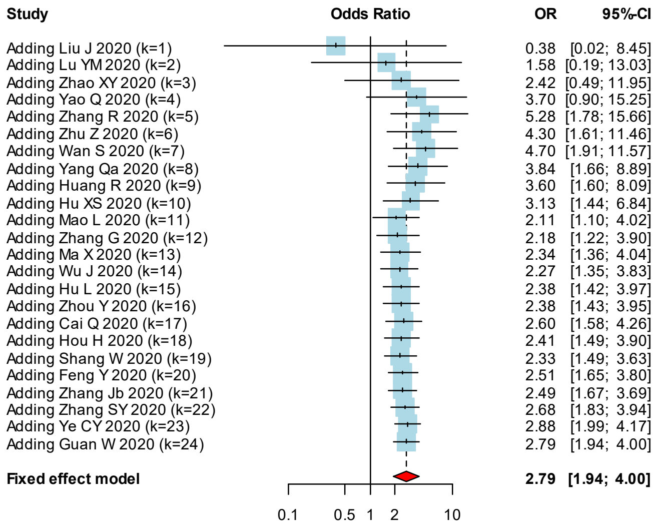

a: Patients' admission date in winter

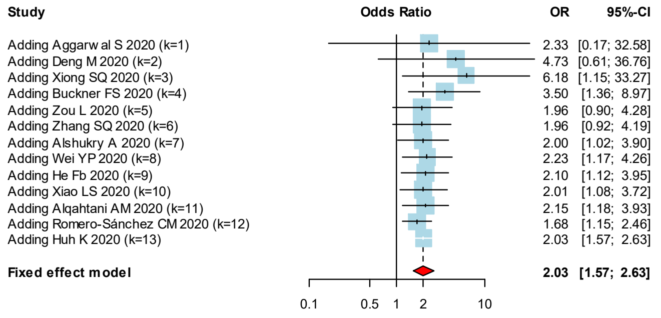

b: Patients' admission date in spring

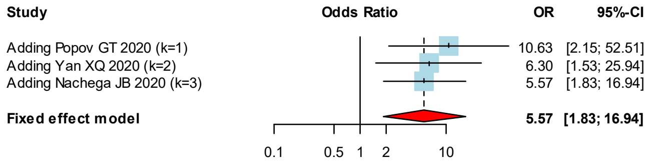

c: Patients' admission date in summer

Supplemental Figure 16: Association between cancer and severity of COVID-19: cumulative meta-analysis

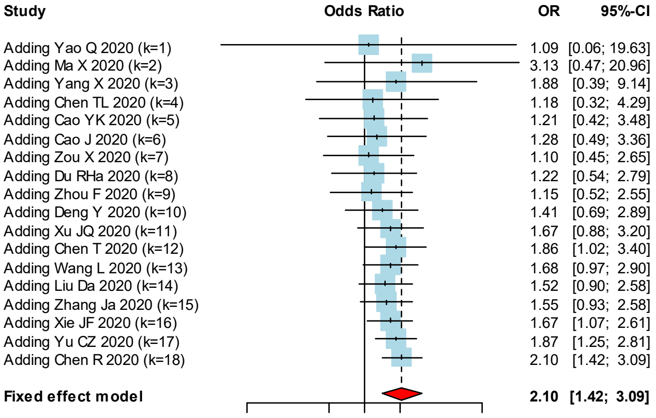

a: Patients' admission date in winter

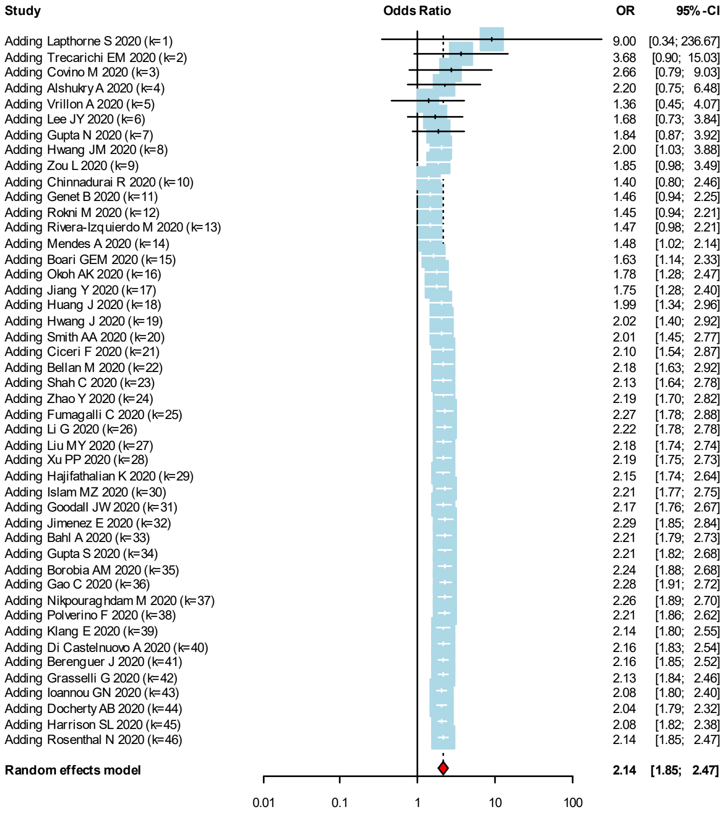

b: Patients' admission date in spring

Supplemental Figure 17: Association between cancer and mortality of COVID-19: cumulative meta-analysis

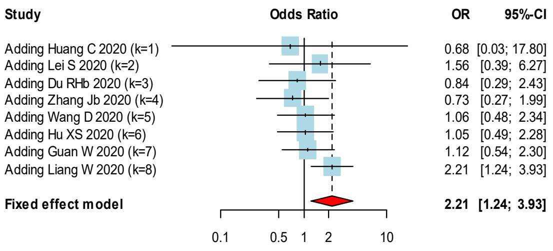

a: Patients' admission date in winter

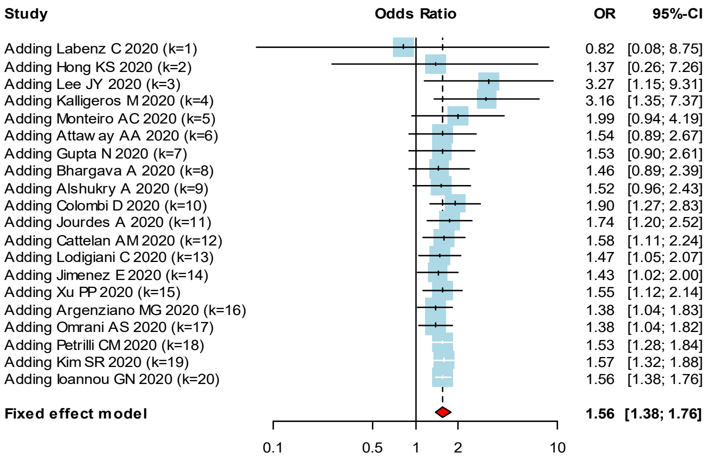

b: Patients' admission date in spring

Supplemental Figure 18: Association between cancer and ICU admission of COVID-19: cumulative meta-analysis
